# Gold Nanoparticle Interference Study during the Isolation, Quantification, Purity and Integrity Analysis of RNA

**DOI:** 10.1371/journal.pone.0114123

**Published:** 2014-12-03

**Authors:** Natasha M. Sanabria, Melissa Vetten, Charlene Andraos, Kailen Boodhia, Mary Gulumian

**Affiliations:** 1 Toxicology and Biochemistry Department, NIOH, Johannesburg, South Africa; 2 Haematology and Molecular Medicine, WITS, Johannesburg, South Africa; University of Helsinki, Finland

## Abstract

Investigations have been conducted regarding the interference of nanoparticles (NPs) with different toxicological assay systems, but there is a lack of validation when conducting routine tests for nucleic acid isolation, quantification, integrity, and purity analyses. The interference of citrate-capped gold nanoparticles (AuNPs) was investigated herein. The AuNPs were added to either BEAS-2B bronchial human cells for 24 h, the isolated pure RNA, or added during the isolation procedure, and the resultant interaction was assessed. Total RNA that was isolated from untreated BEAS-2B cells was spiked with various concentrations (v/v%) of AuNPs and quantified. A decrease in the absorbance spectrum (220–340 nm) was observed in a concentration-dependent manner. The 260 and 280 nm absorbance ratios that traditionally infer RNA purity were also altered. Electrophoresis was performed to determine RNA integrity, but could not differentiate between AuNP-exposed samples. However, the spiked post-isolation samples did produce differences in spectra (190–220 nm), where shifts were observed at a shorter wavelength. These shifts could be due to alterations to chromophores found in nucleic acids. The co-isolation samples, spiked with 100 µL AuNP during the isolation procedure, displayed a peak shift to a longer wavelength and were similar to the results obtained from a 24 h AuNP treatment, under non-cytotoxic test conditions. Moreover, hyperspectral imaging using CytoViva dark field microscopy did not detect AuNP spectral signatures in the RNA isolated from treated cells. However, despite the lack of AuNPs in the final RNA product, structural changes in RNA could still be observed between 190–220 nm. Consequently, full spectral analyses should replace the traditional ratios based on readings at 230, 260, and 280 nm. These are critical points of analyses, validation, and optimization for RNA-based techniques used to assess AuNPs effects.

## Introduction

Plasmonic engineered nanoparticles (NPs) are popular in consumer- and medical-based industries due to their unique surface characteristics. However, there is a growing concern regarding NP toxicity. Identifying the toxicity of NPs is, thus, critical given increased exposure and it has become increasingly important to validate assay parameters for techniques used to determine cyto- and genotoxicity. The toxicity of NPs is often determined using conventional colorimetric and optical high-throughput toxicity systems that rely on absorbance, luminescence or fluorescence signals. However, NPs themselves may interfere with these assay mechanisms [1], thus producing inaccurate results. Overall, there is a lack of assay validation when conducting research with NPs, especially with regard to routine tests for nucleic acid quantification and purity analyses. In addition, toxicity results should be interpreted with caution when using conventional systems, where systems that rely on dyes or optical devices should be avoided. Instead, methods based on label-free technologies should be implemented [2].

Gene expression assays rely heavily on RNA with excellent quality, as emphasized in the MIQE guidelines [3]. Excellent RNA quality refers to an intact RNA sample without visible signs of degradation as indicated by gel electrophoresis, or, a high yield of RNA that is also free of contaminants from the isolation procedure and cellular debris after cell lysis (e.g. DNA, protein and salts) as determined via absorbance ratios. Therefore, the isolation, quantification, purity and integrity analyses are critical points for RNA-based techniques. The most frequently used method, as well as being the least expensive, involves the use of a Nanodrop UV-Vis spectrophotometer to measure absorbance. As originally proposed by Warburg and Christian [4], the A_260_/A_280_ ratio measures the level of protein contamination, and the A_260_/A_230_ ratio indicates whether or not contaminants from the isolation procedure are in the sample (e.g. guanidine salts, EDTA, phenol and carbohydrates) [5]. In addition to determining the yield and purity of RNA, the other key factor for determining the success of the isolation procedure is the RNA integrity. Traditionally, this is determined by assessing the intensity of the ribosomal RNA (rRNA) bands using agarose gel electrophoresis, where eukaryotic cells usually display the 28S and 18S bands.

The UV absorbance of the chromophores that are inherent to nucleic acid structure must be considered when analyzing DNA or RNA using spectrophotometry. Chromophores are chemical groups that absorb UV-visible radiation at a specific wavelength without influencing the other groups in the molecule. Amides (O = C-NH_2_) and amines (-C-NH_2_) are found in the nitrogenous bases of the nucleotides of nucleic acids (both DNA and RNA). Phosphodiester linkages form between nucleotides and constitute the backbone of the molecule. Occasionally amino acids, which contain amino and carboxyl groups, may also precipitate with the final nucleic acid samples after the isolation procedure. Rather than only focusing on the traditional wavelengths, it is important to be aware of all the possible peaks that could be observed during spectrophotometry-based analyses of nucleic acids. An example includes where it was reported that, in addition to the well-known band at 259-260 nm, RNA possessed another stronger absorption band in the far ultraviolet range at 188 nm [6]. These authors found that the spectrum was quite similar to that previously reported for a range observed between native DNA and denatured DNA (at 90°C). They concluded that this behavior conformed to the nature of RNA, which also has a helical character. Another example reported that a π electron system attributed to the sugar-phosphate moiety of the bases in DNA [7], [8], [9]. Absorption bands are pronounced for conjugated π-bond systems, where the π-bond represents a covalent bond formed by the sideways overlap of two ρ-orbitals, e.g. C = C (∼185 nm) and C = N (∼190 nm) double bonds. In addition to the sugar-phosphate moiety, ribose phosphate and deoxyribose (only) were shown to exhibit an increase in absorption spectra below 190 nm [8]. Other factors that should also be considered would be, for example, the presence of the 2'-hydroxyl group found in RNA (in the conformational flexible regions of the molecule not involved in formation of a double helix), where it can chemically attack the adjacent phosphodiester bond to cleave the backbone [10]. In addition, since RNA is charged, metal ions such as Mg^2+^ are needed to stabilize many secondary and tertiary structures within the RNA [11]. The functional form of single-stranded RNA molecules, as also seen in proteins, frequently requires a specific tertiary structure. The scaffold for this structure is provided by secondary structural elements, e.g. hydrogen bonds within the molecule. This leads to several recognizable "domains" of secondary structure, e.g. hairpin loops, bulges, and internal loops [12]. Therefore, full spectrum analyses were performed experimentally herein in order to identify any possible structural changes, which would result in hypochromism (a decrease of peak absorbance) or wavelength shifts.

Considering the chemical compounds and structures of nucleic acids, the linkages between the nucleotides and the additional interactions that stabilize the molecules as described above, it is possible that NPs may interfere at numerous points in the analyses of nucleic acids. Firstly, NPs may interfere with the assay where it measures absorbance at various wavelengths. Secondly, NPs may directly interact with the tertiary structure of nucleic acids and result in changes in the chromophores, e.g. AuNPs may cause a decrease at 260 nm. Therefore, if one were to take into account the proposed assay interference effect of NPs [1], this would result in quantification and purity errors (via absorbance measurements) and obscured integrity validation (via electrophoresis) of the RNA used for gene expression studies.

The current study was initiated in order to determine if any AuNP interference could occur during the isolation, quantification and integrity analyses of RNA obtained from the BEAS-2B human cell line. This was assessed by analyzing the interaction and interference of AuNP with either pure RNA, or, with all the components used during the isolation process. The results presented herein should contribute towards developing a validation procedure that may be used for NP assay interference assessment. Therefore, the questions to be answered are whether or not AuNPs bind to reagents and columns used in the RNA isolation procedure, which may introduce structural changes and subsequently influence the (i) spectrophotometric analyses (ii) electrophoresis analyses (iii) isolation and analyses used for standard gene expression studies. The aim was achieved since an AuNP-spiked sample decreased spectrophotometry-based RNA quantification between 220–340 nm, and interfered with the purity analysis at 230, 260 and 280 nm. In addition, a 24 h treated sample showed slightly discernible effects between 190–220 nm. HSI using CytoViva Dark field microscopy did not detect any AuNP spectral signatures in the isolated RNA from treated cells and the fate of the AuNPs was tracked by UV-Vis absorbance measurements of the eluted flow-through.

## Materials and Methods

An overview of the entire experiment is summarized and provided as supplementary information (see Data S1).

### 1) Synthesis of AuNPs

The AuNPs were 14 nm in size and prepared with sodium citrate as the reducing agent, where trisodium citrate aqueous solution (10 mL, 17 mM) was added to 180 mL (0.3 mM) of boiling HAuCl_4_.3H_2_O aqueous solution [13], [14]. The mixture was boiled under reflux for 15 min and allowed to cool to room temperature. The resultant citrate-capped AuNP suspension, which was deep red in color, was stirred overnight at room temperature. The AuNP suspension was filtered using a 0.25 µm sterile syringe filter (Acrodisc 25 mm PF, 0.2 µm; non-pyrogenic) before use. The synthesis was performed under sterile conditions. Tetrachloroaurate (HAuCl_4_.3H_2_O) and trisodium citrate (Na_3_C_6_H_5_O_7_.2H_2_O) were purchased from Sigma Aldrich (USA) and used without further purification. This AuNP was fully characterized (see Data S2) and the results published [2].

### 2) Cell culture

The bronchial epithelial human cell line, BEAS-2B, was obtained from Sigma Aldrich (catalogue number 951 95102433), originally from the European Collection of Cell Cultures (ECACC), operated by the Health Protection Agency Culture Collections (HPACC). The BEAS-2B cell line was maintained under standard culturing conditions (37°C, 5% CO_2_ in a humidified environment), in RPMI-1640 medium with L-glutamine (Lonza, Germany), 10% heat-inactivated fetal bovine serum (FBS, Lonza, Germany), 100 units Penicillin per mL culture media and 100 µg Streptomycin per mL culture media (Lonza, Germany), hereafter referred to as RPMI culture medium. The cell monolayer (at 80–90% confluence) was washed with Dulbecco's Phosphate Buffered Saline (DPBS, Lonza, Germany), and harvested by incubation with a 0.5% trypsin/EDTA (Sigma Aldrich, USA) solution. The cells were collected by centrifugation at 200 × *g* for 5 min. The supernatant was discarded and the cells were subsequently re-suspended in 10 mL RPMI culture media. The cell viability was determined using trypan blue exclusion method (Invitrogen, USA). Thereafter, the cells were routinely sub-cultured every 3–4 days at approximately 6 × 10^3^ cells/cm^2^. Experiments were performed with BEAS-2B bronchial human cells between passages 15 to 20.

### 3) Treatment

BEAS-2B cells were seeded at approximately 3 × 10^4^ cells/cm^2^ in a 75 cm^2^ flask and allowed to proliferate for 24 h before treatment. Cells were treated with a non-cytotoxic concentration of 1 nM AuNPs, as determined by cell impedance analyses [2]. Untreated cells were used to prepare the different controls as specified below.

### 4) RNA isolation

Total RNA was isolated using the RNeasy plus mini kit (Qiagen, GmbH), according to the manufacturer's instructions. In addition, the QIAshredder spin columns (Qiagen, GmbH) were used to homogenize the samples (see Data S1). The RNAprotect stabilizing solution was used for both control and 24 h-treated samples due to time constraints resulting from the handling procedure (see Data S3). Briefly, following trypsinization and harvesting of the cells, RNAprotect solution was added to intact cells to stabilize the RNA. The RNA lysis buffer with guanidine thiocyanate (RLT) was added and vortexed to lyse the cells. The cell lysate was passed through a QIAshredder column to aid homogenization, followed by a gDNA Eliminator column to remove genomic DNA. Ethanol was added and the sample loaded onto an RNeasy MinElute column, where RNA binds to the column and contaminants were washed away during subsequent wash steps with the RNA wash buffer (RW1) and the RNA ethanol-based buffer (RPE). Finally, RNA was eluted with RNase-free water. Using these same steps, RNA was also isolated from 24 h AuNP treated BEAS-2B cells.

The RNA obtained from untreated cells served as the control. The AuNP-spiked samples represent different variations of positive controls. The post-isolation spiked sample was prepared by using the untreated control RNA, which was spiked with various percentages of AuNP. The 25% NP spike had a final concentration (FC) of 0.75 nM. The 50%, 75% and 100% each had an FC of 1.5 nM, 2.25 nM and 3 nM, respectively. The co-isolation spiked sample was prepared by using the untreated cells (in the RNAprotect solution), which were spiked with various volumes of AuNPs, prior to cell lysis and RNA isolation. However, there was no extended incubation time similar to the 24 h AuNP-treated samples. The 25 µL NP spike had an FC of 0.1667 nM. The 50 µL and 100 µL spikes each had an FC of 0.333 and 0.667 nM, respectively. The RNA obtained from untreated cells was also used to prepare an additional spiked sample, where the sample had a constant amount of RNA, but had variable amounts of AuNP. This was a variation of the post-isolation spiked sample, where the untreated control RNA was first diluted and then kept at a constant amount of 20 ng, but still spiked with various percentages of AuNP. Therefore, the 25% NP spike had a constant amount of 20 ng RNA with an AuNP FC of 0.75 nM. The 50%, 75% and 100% each had an AuNP FC of 1.5 nM, 2.1 nM and 3 nM, respectively.

### 5) RNA quantification

The RNA was analyzed on a Nanodrop 2000c UV-Vis Spectrophotometer, according to the manufacturer's instructions. Traditional UV-Vis spectra were obtained from 220 to 350 nm, where nucleic acid quantification was determined at 260 nm. The purity of each sample was determined using the A_260_/A_280_ and A_260_/A_230_ ratios. Additional full spectra were obtained from 190 to 840 nm. Post and co-isolation spiked samples were compared to the untreated control and statistical analyses were performed using the Student's T-test (paired; one-tailed distribution). The interpretation of the *p*-value was based on a significance level of 5%, where: p≤0.005 referred to a very strong presumption against the null hypothesis, which was designated as “very significant”; 0.005≤p≤0.01 referred to a strong presumption against the null hypothesis, which was designated as “significant”; 0.01≤p≤0.05 referred to a low presumption against the null hypothesis, which was designated as “low significance”; p>0.05 referred to a no presumption against the null hypothesis, which was designated as “not significant”.

### 6) Agarose gel electrophoresis

For RNA samples, a 10 µL aliquot was added to 2 µL of a 6xOrange Loading dye containing Orange G and Xylene Cyanol (Fermentas). This was loaded onto a 1% agarose gel and subjected to electrophoresis at 100V, whilst being submerged in 89 mM Tris-borate and 2 mM EDTA at pH 8.3 (TBE) buffer (Sigma Aldrich, USA), which was stained with 10 µg/mL ethidium bromide. Images were obtained using GeneSys software version1.3.3.0 on a Syngene G:Box instrument (grey-scale).

### 7) CytoViva dark field microscopy and HSI

For uptake studies, cells were seeded in 8-well Millicell EZ slides (Millipore, Germany) prior to treatment. Following incubation, cells were washed with culture media, followed by three washes with DPBS (Lonza, Germany). Cells were fixed at 4°C with 4% formalin in Tris/HCl buffer, at pH 7.4, for 15 min. Slides were washed once with DPBS and air-dried. Cover-slips were immobilized onto slides with Kaiser's gelatin (Merck, Germany). Dark field images were captured at 60× magnification using the CytoViva 150 Unit integrated onto the Olympus BX43 microscope. Images were acquired using a Dagexcel ×16 camera and the associated software.

To investigate the fate of the AuNPs, a drop of either AuNP in Milli-Q water or the isolated RNA from AuNP-treated cells was placed onto a Millicell EZ slide and allowed to dry. Samples were visualized using dark-field microscopy, at 60× magnification and using the CytoViva150 unit integrated onto the Olympus BX43 microscope. Images were acquired using a DAGExcel ×16 camera and software. HSI was performed at 60× magnification using the HSI system 1.1 and ENVI software. A spectral library of the 14 nm AuNPs was created by randomly selecting spectra of AuNPs, where each spectrum of the library represents a single pixel obtained from the HSI scan. In order to investigate the presence of the AuNPs in the RNA sample, the image classification algorithm, spectral angle mapper (SAM), was performed using ENVI software to map the spectral libraries onto the scans. Therefore, the spectral profile collected was representative of a spectral library of the AuNP onto the HSI scan of the RNA.

### 8) Tracking the fate of the NPs

To investigate the fate of the AuNPs throughout the RNA isolation procedure, samples were retained at various steps. The samples collected were from key points in the harvesting and RNA isolation procedure, namely, (1) the cell culture media in which the cells were treated, (2) the PBS used to wash the cells prior to trypsinization, (3) the supernatant following centrifugation of the trypsinized cells, (4) RNAProtect solution supernatant, (5) RLT buffer and ethanol following sample loading onto RNeasy MinElute column, (6) Buffer RW1 following wash step, (7) Buffer RPE following first wash step, and (8) Buffer RPE following second wash step. Full range spectra were obtained (from 190 to 840 nm) on a Nanodrop 2000c UV-Vis Spectrophotometer, according to the manufacturer's instructions. Nuclease free water was used as a blank for all the different samples. For each eluent analyzed (e.g. the first “media” eluent), the absorbance reading of the media eluted from the untreated control represented the background reading, and, was subtracted from the absorbance reading of the media eluted from the AuNP-treated sample, and, the resultant difference in absorbance reading was plotted, where:

Difference Abs-media  =  (Abs-media from AuNP-treated) - (Abs-media from untreated control)

## Results

### 1) Spectrophotometric-based RNA quantification of spiked samples

**Figure 1:**
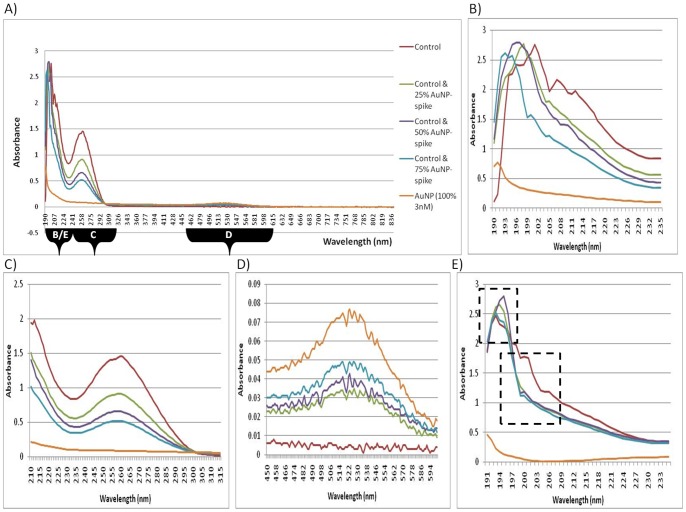
UV-Vis spectroscopy of RNA from post-isolation spiked samples. (**A**) Full spectrum absorbance analyses of artificial AuNP-spikes, measured at wavelengths from 190–840 nm. (**B**) Peak shifts of absorption spectra of artificial AuNP-spikes, measured at wavelengths from 190–235 nm. (**C**) Traditional absorbance spectrum analyses of artificial AuNP-spikes, measured at wavelengths from 220–340 nm. (**D**) Absorption spectra of artificial AuNP-spikes, measured at wavelengths from 450–600 nm. (**E**) Spiked samples containing a constant amount of RNA with variable percentage of AuNPs, indicating the absorption spectra measured at wavelengths from 190–235 nm.

The most common wavelength range tested before gene expression studies is the 220–340 nm range since the absorbance measurements at 230, 260 and 280 nm are used to calculate RNA yield and purity. Therefore, these wavelengths were included for initial studies (Figure 1C). Post-isolation spiked samples showed a concentration-dependent decrease in the absorbance of the peak found at 260 nm in the RNA samples that were spiked with AuNPs. Since RNA yield is calculated based on the absorbance at 260 nm, where the presence of AuNPs caused a decrease in the absorbance reading, a false quantification of the RNA yield was calculated (Table 1).

**Table 1:** Purity analysis of untreated control vs. post-isolation AuNP-spiked RNA samples.

Sample	Without *RNAprotect solution*						With *RNAprotect solution*						Comments
	Average A_260_/A_280_	Std Dev A_260_/A_280_	Average A_260_/A_230_	Std Dev A_260_/A_230_	Average yield compared to control	*p*-Value	Average A_260_/A_280_	Std Dev A_260_/A_280_	Average A_260_/A_230_	Std Dev A_260_/A_230_	Average yield compared to control	*p*-Value	
**Control**	2.053	0.037	1.483	0.210	100%	N/A	2.036	0.045	1.916	0.055	100%	N/A	Good quantity and purity.
**Control + 25% AuNP**	2.053	0.045	1.366	0.118	83.31%	0.029*	2.05	0.045	1.743	0.180	73.70%	0.016*	Reduced yield; no protein contamination; no contaminants from isolation procedure when Protect solution used.
**Control + 50% AuNP**	2.033	0.075	1.333	0.058	57.62%	0.001***	2.05	0.043	1.870	0.105	58.92%	0.001***	Reduced yield; no protein contamination; no contaminants from isolation procedure when Protect solution used.
**Control + 75% AuNP**	2.006	0.050	1.356	0.208	45.72%	0.006**	2.02	0.062	1.763	0.125	36.91%	1.743E-05 ***	Reduced yield; no protein contamination; no contaminants from isolation procedure when Protect solution used.

**Note:** *** p≤0.005; ** 0.005≤p≤0.01; * 0.01≤p≤0.05. **(Std Dev) Standard Deviation.**

The experiment was further expanded upon to include a full spectrum scan in the UV-Vis region, from 190 to 840 nm (Figure 1A). Differences in spectra were noted at approximately 190-220 nm, where peak shifts were observed for post-isolation AuNP-spiked samples (Figure 1B). The shifts occurring in the spiked samples were all in the region of 190 nm and the peak was observed at a shorter wavelength, compared to the control sample. A slight peak was also observed for the AuNP in Milli-Q water (3 nM), in this wavelength region. As the concentration of AuNP increased in the spiked samples, a new and small peak was observed from 500–580 nm (Figure 1D), which corresponds to the absorbance peak observed in the characterization of the AuNPs. It was also interesting to note that as the new peak changed by small increments in amplitude between 500–580 nm, the peak at 260 nm decreased (i.e. the two peaks were related and inversely proportional to each other). Therefore, it is recommended that full spectrum analyses be performed for each type of NP used in order to detect any assay interference.

A variation of the post-isolation spiked sample was included, which consisted of RNA isolated from untreated cells. The RNA was diluted and then kept at a constant amount of 20 ng. This diluted RNA was spiked with AuNP in a similar manner to the post-isolation spiked sample series. Differences in spectra were observed between approximately 190 to 220 nm, where peak shifts were observed for AuNP-spiked samples (Figure 1E). Although the differences were not AuNP concentration dependent, these shifts could be subdivided into two groups, at wavelengths that correspond to C = N double bonds as well as other known chromophores that are present in nucleic acids (Table 2). In addition, the results also correspond to the peak shifts observed for the post-isolation controls (Figure 1B and Figure 1E). A slight peak was observed, again, for the AuNP at approximately 190 nm.

**Table 2:** UV absorption bands for chromophores, adapted from [29].

Chromophore	Formula	Wavelength nm (ε at maximum)*	Relation to RNA samples
Amine	–NH_2_	195 (3000)	Present in purines/pyrimidines, e.g. the nitrogenous bases.
Ester	O = C–O–C	205 (50)	Substitution of Carbon with Phosphor creates the phosphodiester bond (O = P–O–C), which forms the linkage between nucleotides.
Carboxyl	O = C–O–H	205 (60)	Present in amino acids, which may contaminate RNA isolations.

*The extinction coefficient *ε*  =  M_r_.(A/cl), and is related to the relative molecular mass, M_r_.

**Figure 2:**
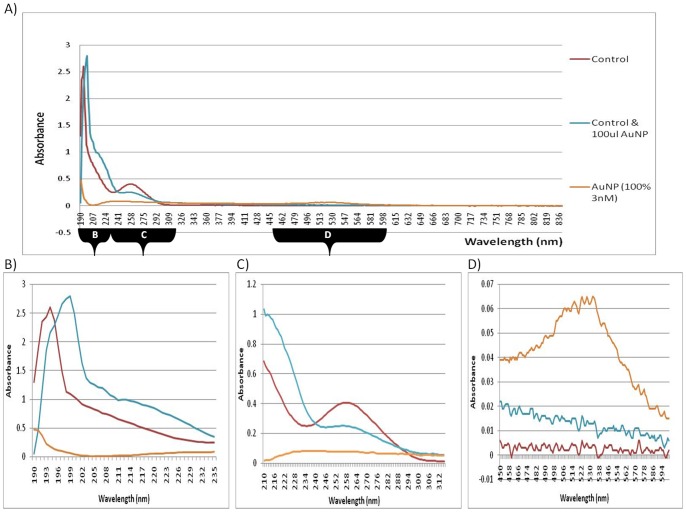
UV-Vis spectroscopy of RNA from co-isolation spiked samples. (**A**) Full spectrum absorbance of artificial AuNP-spikes, measured at wavelengths from 190–840 nm. (**B**) Peak shifts of absorption spectrums of artificial AuNP-spikes, measured at wavelengths from 190–235 nm. (**C**) Traditional absorbance spectrum of artificial AuNP-spikes, measured at wavelengths from 220–340 nm. (**D**) Absorption spectrums of artificial AuNP-spikes, measured at wavelengths from 450–600 nm.

The experiment was expanded upon to include a “co-isolation” step, where various AuNP volumes were added prior to cell lysis and then processed in parallel up to the point of RNA precipitation (Figure 2 and Table 3). It was noted that due to the numerous wash and centrifugation steps involved in the RNA isolation procedure, from the point of cell lysis to RNA precipitation, variable results were obtained for the various concentrations of AuNP used. It was also observed that unusual black precipitates formed as the AuNPs reacted with the reagents and purification columns during the isolation procedure (see Data S3). Therefore, only the co-isolation spiked sample with the 100 µL AuNP spike was used for further comparative purposes.

**Table 3:** Purity analysis of untreated control vs. co-isolation AuNP-spiked RNA samples.

Sample	Without *RNAprotect solution*						With *RNAprotect solution*						Comments
	Average A_260_/A_280_	Std Dev A_260_/A_280_	Average A_260_/A_230_	Std Dev A_260_/A_230_	Average yield compared to control	*p*-Value	Average A_260_/A_280_	Std Dev A_260_/A_280_	Average A_260_/A_230_	Std Dev A_260_/A_230_	Average yield compared to control	*p*-Value	
**Control**	2.063	0.032	1.203	0.349	100%	N/A	2.106	0.015	1.73	0.020	100%	N/A	No contaminants from isolation procedure when Protect solution is used.
**Control + 25 µL AuNP**	2.066	0.020	1.876	0.250	239.6%	0.180	2.066	0.015	1.63	0.503	87.16%	0.024*	Highly variable results obtained.
**Control + 50 µL AuNP**	2.076	0.015	1.326	0.774	200.4%	0.250	2.086	0.046	2.026	0.015	80.03%	0.031*	Highly variable results obtained.
**Control + 100 µL AuNP**	2.016	0.092	1.403	1.117	147.4%	0.338	2.076	0.037	1.576	0.143	93.3%	0.056*	Highly variable results obtained.

**Note:** *** p≤0.005; ** 0.005≤p≤0.01; * 0.01≤p≤0.

**Figure 3:**
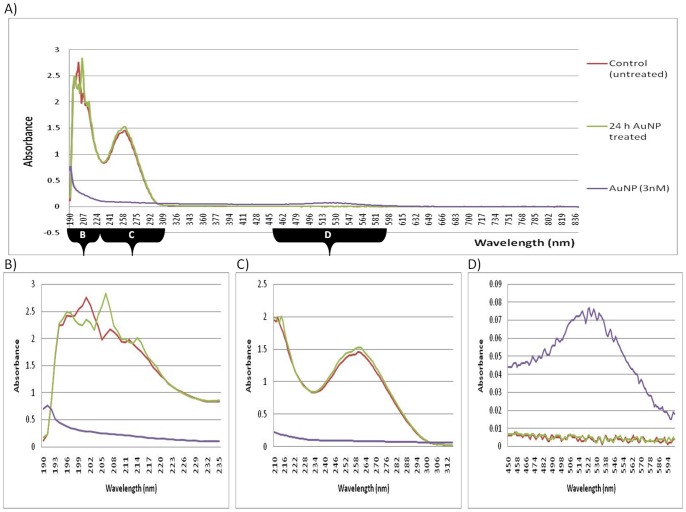
UV-Vis spectroscopy of RNA from 24 h AuNP-treated samples. (**A**) Full spectrum absorbance analyses of RNA obtained from 24 h AuNP-treated cells, measured at wavelengths from 190–840 nm. (**B**) Peak shifts of absorption spectra of RNA obtained from 24 h AuNP-treated cells, measured at wavelengths from 190–235 nm. (**C**) Traditional absorbance spectrum analyses of RNA obtained from 24 h AuNP-treated cells, measured at wavelengths from 220–340 nm. (**D**) Absorption spectra of RNA obtained from 24 h AuNP-treated cells, measured at wavelengths from 450–600 nm.

**Figure 4:**
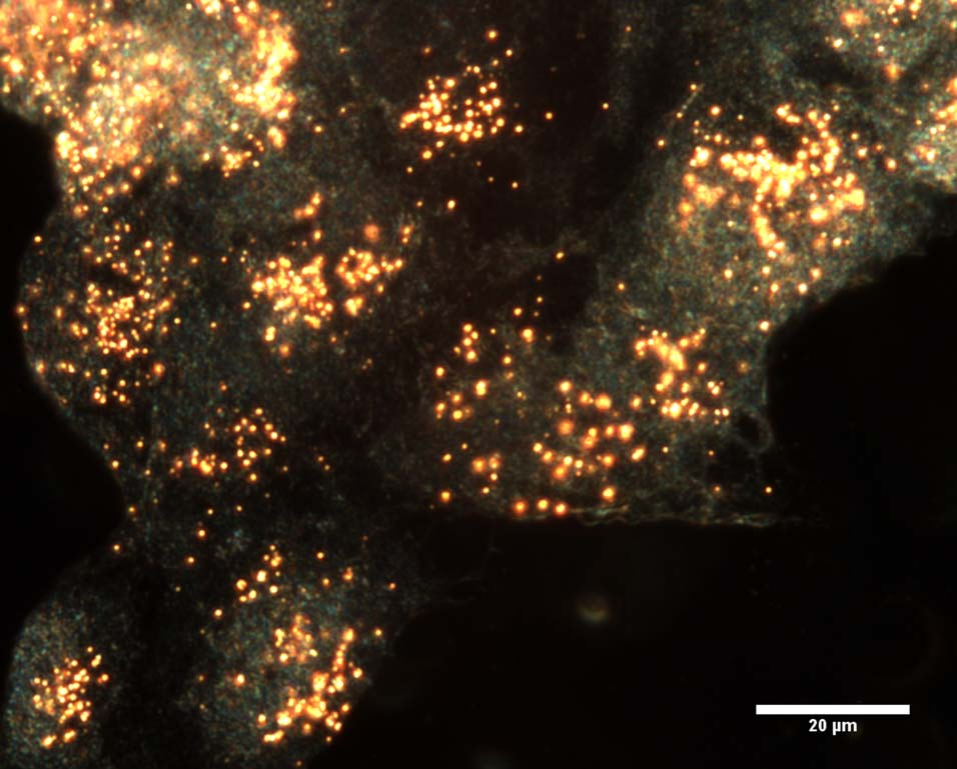
Dark field image at 60× magnification of BEAS-2B cells incubated with 1 nM AuNPs for 24 h.

In Figure 2, the co-isolation sample spiked with the 100 µL AuNP, displayed a peak shift to a longer wavelength in comparison to the control sample. Again, the observed shift was in the 190 nm wavelength region (see chromophores in Table 2). The co-isolation results were similar to the results obtained when analyzing total RNA that was isolated from BEAS-2B cells exposed to a 24 h AuNP-treatment (Figure 3). However, the co-isolation spiked sample had a slightly higher concentration of AuNPs (Figure 2D) than the 24 h treated sample (Figure 4D), which is consistent with the amount of AuNPs that were deliberately added during the co-isolation procedure.

### 2) Spectrophotometry-based RNA quantification of 24 h AuNP-treated samples

The current study also included the assessment of RNA obtained from samples that had been treated with AuNPs for 24 h. Uptake of AuNPs into BEAS-2B cells has been previously observed at 1, 4 and 6 h, with minimal toxicity [2]. However, a high level of uptake of the AuNPs into BEAS-2B cells was observed at 24 h (Figure 4). Therefore, it was decided to use this time point to investigate the effects of the internalized AuNPs on RNA isolation.

A few indications of AuNP interference were discernible in the RNA obtained from 24 h AuNP-treated samples (Figure 3). Although the AuNP did react with the reagents and purification columns in a similar manner to the co-isolation spiked sample, the RNAprotect stabilizing solution appeared to minimize the AuNP interference, where the spectrophotometric-based quantification of RNA showed only slightly discernible interference, and, the absorbance decreased between 220–340 nm (Figure 3C).

Since the initial data focused only on a small wavelength range from 220–340 nm (the most common region tested before gene expression studies), the experiment was further expanded upon to include full spectra in the UV-Vis region (190–840 nm). Differences were observed for the 24 h AuNP-treated sample, at wavelengths other than the traditional measurements, i.e. peak shifts were observed at 190–220 nm. In Figure 2, the co-isolation spiked sample (with the 100 µL AuNP spike) displayed a peak shift to a longer wavelength. In Figure 3B, the 24 h AuNP-treated sample displayed a peak shift in the region from around 190 nm to 205 nm and resulted in a longer wavelength compared to the control sample. Therefore, both forms of exposure to AuNP had similar shifts in absorbance. However, the co-isolation spiked sample had a higher concentration of AuNPs, whereas the 24 h-treated samples had an undetectable amount of AuNPs in the final precipitated product (Figure 3D). Therefore, the only AuNPs that could have been present and been in contact with the RNA would have been those that had migrated across the cell membrane and were already within the cells during the RNA isolation. Collectively, both the co-isolation spiked sample results and the 24 h AuNP-treated results then demonstrate the possible interference effect of AuNPs when they are present during cell lysis and a subsequent nucleic acid isolation procedure. This is despite the fact that at 500–580 nm, the peak that was previously seen in the post-isolation spiked samples (Figure 1), was not observed in this 24 h AuNP-treated sample (Figure 3D). This indicates that AuNPs were effectively removed from the 24 h AuNP-treated sample, which was further confirmed by CytoViva dark field microscopy and HSI (see below).

### 3) Spectrophotometry-based RNA purity analysis of spiked samples

The ratios obtained from the 230 nm, 260 nm and 280 nm absorbance readings are traditionally used to assess RNA purity (Tables 1 and 3). The ideal ratio for RNA for A_260_/A_280_ (an indication of protein contamination) is between 1.8 and 2.1. The ideal ratio for RNA for A_260_/A_230_ (indicating guanidine salts, EDTA, phenol and carbohydrate contaminants), is ideally above 1.5 (2.0–2.2). A decrease in the absorbance spectrum, from 220 nm to 340 nm, (Figure 1 and Table 1 was observed for post-isolation samples that were spiked with AuNP (3 nM; refer to FC in methods section). In addition to quantification errors at 260 nm, the quenching effect could cause errors in purity analyses using A_260_/A_280_ and A_260_/A_230_ ratios (see Data S4). The co-isolation spiked samples produced variable results. It should be noted that the spiked samples have a considerably higher amount of AuNP present during the RNA isolation procedure compared to the 24 h treated samples. This occurred since the AuNPs associated with the culture media during the 24 h treatment and were separated from the sample prior to cell lysis (discussed below). These are critical points in RNA analyses for subsequent RNA-based techniques.

### 4) Electrophoresis-based RNA integrity analysis of spiked samples

**Figure 5:**
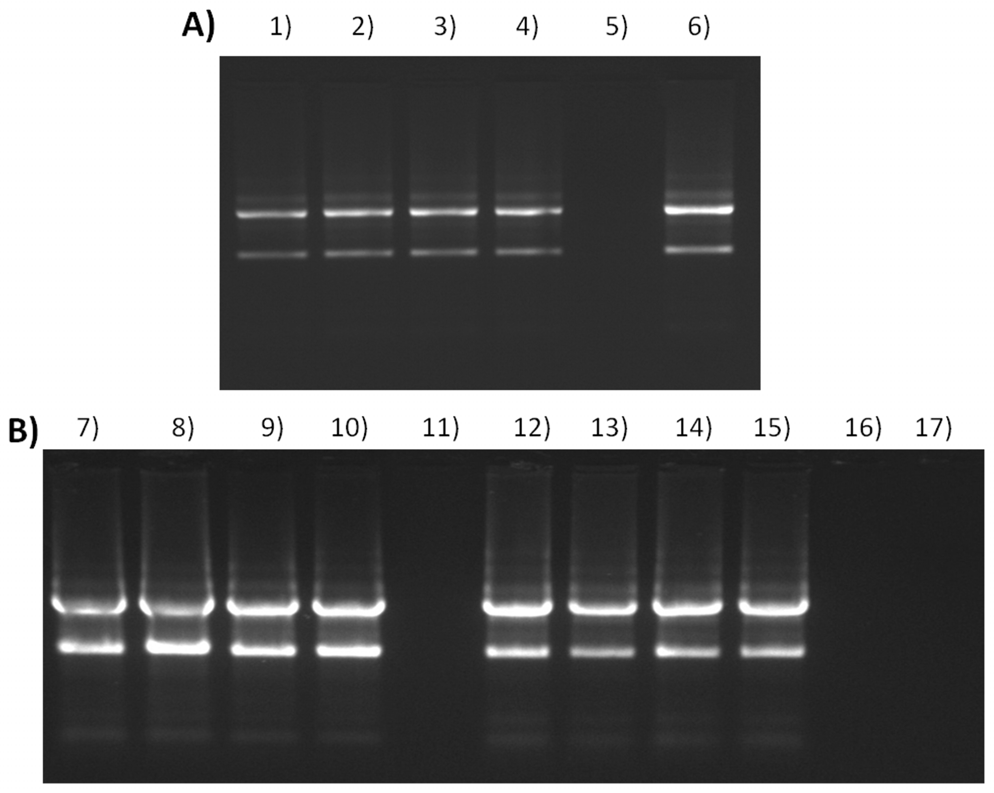
RNA integrity analysis. (**A**) Post-isolation spiked samples. (**B**) Co-isolation spiked samples. Lanes: (**1**) RNA untreated control. (**2**) RNA untreated control with RNAProtect solution +25% AuNP-spike. (**3**) RNA untreated control with RNAProtect solution +50% AuNP-spike. (**4**) RNA untreated control with RNAProtect solution +75% AuNP-spike. (**5**) AuNP (3 nM) with 6xOrange Loading dye (Fermentas). (**6**) The 24 h AuNP-treated RNA with RNAProtect solution. (**7**) RNA untreated control. (**8**) RNA untreated control +25 µL AuNP-spike. (**9**) RNA untreated control +50 µL AuNP-spike. (**10**) RNA untreated control +100 µL AuNP-spike. (**11**) Unloaded lane. (**12**) RNA untreated control with RNAProtect solution (**13**) RNA untreated control with RNAProtect solution +25 µL AuNP-spike. (**14**) RNA untreated control with RNAProtect solution +50 µL AuNP-spike. (**15**) RNA untreated control with RNAProtect solution +100 µL AuNP-spike. (**16**) Unloaded lane. (**17**) AuNP (3 nM) with 6xOrange Loading dye (Fermentas).

Horizontal/submerged 1% agarose gel electrophoresis was performed in TBE buffer. The resulting RNA fragments were visualized using a 10 µg/mL Ethidium Bromide stain. In eukaryotic cells, the 28S (upper) band should be twice the intensity of the 18S (lower) band [15], see lanes 12 to 15 in Figure 5. Smearing between the 28S and 18S bands is considered normal since most of the mRNA migrates to this region within the gel. However, smearing far below the 18S band, or, loss of either of the bands in addition to accumulation of degraded RNA near the bottom of the gel, is indicative of a loss of RNA integrity.

There was no discernible difference between the banding patterns of the untreated control RNA to the AuNP post- or co-isolated spiked samples (see lanes 1–4, 7–10 or 12–15 in Figure 5). Therefore, electrophoresis was not sensitive enough to detect any changes in the RNA, nor did this specific 14 nm AuNP influence the assay (see lanes 5 and 17 in Figure 5). In addition, the AuNP on its own does not appear to retard migration of the sample buffer dyes. However, any of the RNA samples that contained this AuNP, did react with sample buffers containing Ficoll 400, where the sample density changed and influenced sample loading into the wells of the gel. A sample buffer consisting of mainly Tris-HCl, glycerol and EDTA appeared to be more suitable.

### 5) The use of RNAProtect stabilizing solution during RNA isolation

A commercially available solution normally used to maintain RNA integrity, during storage or processing, was tested in order to determine the putative reaction with the AuNP. Although distinct differences were expected, only slight improvements were observed whilst using this protection/stabilizing solution. For example, a slight shielding from the effects of the added AuNP-spike to the control RNA was observed (as indicated by the A_260_/A_280_ and A_260_/A_230_ ratios in Table 1). Occasionally a decrease in RNA yield was observed whilst using the solution, when compared to the original method (see Data S4). In addition, the only improvement in RNA integrity visible on the gel was that the 28S (upper) band was twice the intensity of the 18S (lower) band, see lanes 12 to 15 in Figure 5. Another factor to consider was that the use of this solution required additional optimization steps. It also occasionally produced (pink) precipitates in the co-isolated AuNP-spiked RNA samples (see Data S3). The main advantage to using the solution was observed when isolating RNA from 24 h AuNP-treated cells, where the yield in Figure 3C, which is determined at 260 nm, was similar to that of the control. In addition, the 24 h treated sample (Figure 5, lane 6) displayed the sought after upper 28S band of twice the intensity of the lower 18S band.

### 6) CytoViva dark field microscopy and HSI

**Figure 6:**
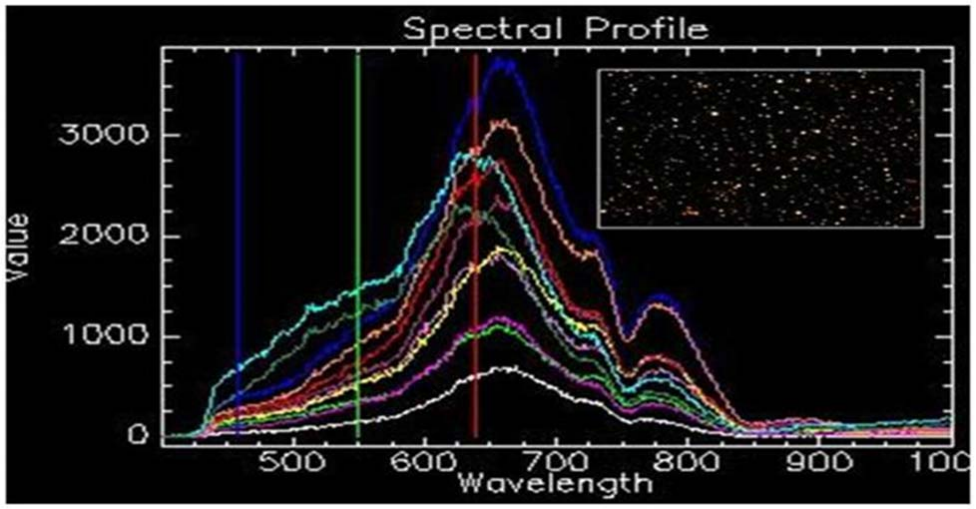
The spectral profile of 14 nm citrate-capped gold nanoparticles. (**A**) The profile was collected from ten randomly selected nanoparticles (singularly dispersed and aggregated AuNPs). (**B**) The insert indicates the image obtained using DAGE software.

CytoViva dark field microscopy was used in conjunction with HSI on the isolated RNA, in order to determine if AuNPs were co-precipitated during the cell harvesting and RNA isolation procedure. Figure 6 shows ten spectral profiles, collected from randomly selected particles (singularly dispersed and aggregated AuNPs), and each represents a single pixel obtained from the HSI scan. The SAM analysis tool uses the unique spectral profile of a known material for its identification in an image captured using hyperspectral imaging. Identified pixels are mapped to the known spectra, irrespective of light intensity, which can then be used as a spectral library for the 14 nm citrate-capped AuNP to confirm the presence of the AuNPs in a HSI scan of an unknown sample (the HSI scan of isolated RNA) using SAM. SAM was employed to verify the presence or absence of the particles in the isolated RNA. The spectral library collected from the AuNPs (Figure 6) was mapped against the HSI scan of isolated RNA, with no mapped spectra indicated.

### 7) Tracking the fate of NP during cell culturing and RNA isolation

**Figure 7:**
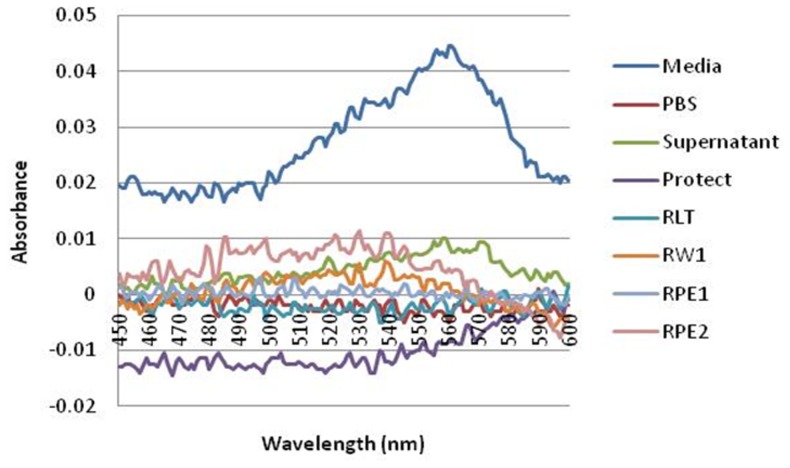
The fate of the AuNPs during cell harvesting and RNA isolation. The absorbance of the control sample was subtracted from the AuNP-treated sample and the difference was plotted.

The decrease in UV-Vis absorbance, or AuNP interference, observed with the spiked post-isolation samples was not observed in the RNA isolated from 24 h AuNP-treated BEAS-2B cells. It is proposed that the methodology used for the harvesting of the cells and isolation of the RNA removed the AuNPs from the sample. During cell harvesting of an adherent cell line (BEAS-2B), the culture media is removed from the cells, and then washed with PBS, before being trypsinized. The RNA isolation procedure that followed included wash and centrifugation steps. The AuNPs were tracked through the eluted flow-through or discarded waste (eluents) from all the wash steps produced during the RNA isolation procedure for BEAS-2B cells treated for 24 h. The citrate-capped AuNP has an absorbance at 519 nm. However, it was decided to plot the difference in absorbance readings between the untreated control and the 24 h treated sample, from 450 to 600 nm, per eluent (Figure 7). It was observed that the AuNPs formed visible precipitates at three different stages, namely the first “media-only’ eluent, the cell pellet (which formed black precipitates) and the supernatant eluent containing media and trypsin. In addition, the AuNPs that were visually observed to associate with the cells were subsequently trapped by the QIAshredder spin column during cell lysis (see Data S3).

## Discussion

This study was initiated in order to determine if any AuNP interference occurred during the isolation, quantification and integrity analyses of RNA obtained from the BEAS-2B human cell line. This was achieved by assessing the interaction and interference of AuNP with either the isolated RNA sample, or, during the isolation process. This particular AuNP was fully characterized [2], and used at the various concentrations as indicated. Spiked samples were used to identify the putative AuNP interference at the various stages such as, (i) interference with the buffers, columns and washes used during the isolation, (ii) interference with the absorption quantification readings and purity analyses, (iii) interference with the electrophoresis-based integrity analyses, (iv) interference with the protective solutions used to stabilize cells/tissues before RNA isolation and finally the most important stage (v) interference with the structural integrity of the isolated RNA. The study was then expanded to assess RNA obtained from cells treated with a non-cytotoxic concentration (1 nM) of the same AuNP for 24 h. HSI was used to determine whether or not AuNPs precipitated with the RNA. Full UV-Vis spectra were used to track the fate of the NPs during cell harvesting and RNA isolation, by testing the various discarded material, flow-through and eluents. The novel findings are discussed in detail below, as indicated.

### 1) Comparison between spiked samples and the 24 h AuNP-treated samples

In a recent study, spectral analysis was used to elucidate details regarding the structure and extent of denaturation of DNA induced by weakly charged cationic NPs [16]. It was, thus, proposed that if the AuNP tested herein did interfere with the isolation, quantification, purity and integrity analysis of RNA, then it would be observed via UV-Vis absorbance.

It is important to note the shared differences and similarities between the samples. For example, both the post- and co-isolation spiked samples had a higher concentration of AuNP than the 24 h treated sample since the spiked samples contained AuNPs that were deliberately added to the RNA sample (at various percentages and volumes). The treated sample only contained a non-cytotoxic concentration [2], of 1 nM AuNPs (14 nm in size), which had migrated into the cell (Figure 4). However, even between both (similar) spiked samples, the post-isolation spiked sample exceeded the non-cytotoxic concentration, whereas the co-isolation samples did not. Another difference was the extent of exposure between the spiked and treated samples, where the post-isolation spiked sample was only exposed to AuNPs after the pure RNA had already been isolated. The co-isolation spiked sample and the treated samples were similar, where both were collected after the AuNPs were present and exposed to the buffers and columns used during the isolation procedure.

Table 1 summarizes the difference between the untreated control and the post-isolation spiked samples. “Significant” to “very significant” changes were determined for the purity ratios between the RNA end-products spiked with either 50% or 75% AuNP. A decrease in the absorbance in the region of 220–340 nm was not observed in the 24 h treated sample, despite the fact that it was observed in samples that were deliberately spiked with AuNP. The lack of absorbance quenching in the 24 h treated sample was most probably due to the low concentration of AuNPs present in the treated cells compared to the spiked sample. In addition, the post-isolation spiked samples underwent a slight wavelength shift towards a lower wavelength between 190–220 nm, but the co-isolation and the 24 h treated samples both underwent a slight wavelength shift to a higher wavelength. The AuNP (3 nM) also showed a slight peak at this lower wavelength and most probably explains the shift observed in the post-isolation sample. The characteristic 450–600 nm AuNP peak was also lacking in the 24 h treated samples and, subsequently, prompted the idea that the shift to a higher wavelength between 190–220 nm (as also observed in the co-isolation spiked sample), could be an indicator of RNA damage that was caused by AuNPs being present during cell lysis, even though the AuNPs may not necessarily be present in the final RNA product. It was also noted that the optimized method used to isolate this RNA was able to prevent AuNP co-precipitation (as verified by CytoViva dark field microscopy). Therefore, although the precipitated sample may not contain AuNPs, the isolated RNA obtained from treated cells might already be compromised during isolation and, thus, would not be fit for further analyses, e.g. gene expression studies.

Table 3 summarizes the difference between the untreated control and the co-isolation samples. “Low” to “no significance” was determined for changes in the RNA end-product obtained after isolation, even though known amounts of AuNP had been present during the process. The traditional measurements at 230, 260 and 280 nm for nucleic acid analyses were clearly not sufficient for nanoparticle-related research. Only full spectrum analyses detected assessment interference caused by AuNPs during the isolation, quantification, purity and integrity analyses of RNA.

### 2) Causes of the wavelength shifts observed in Nucleic acid samples treated with NPs

Spectrophotometry-based analysis of protein was previously performed by detection of peptide/amide bonds (covalent bonds between amino acids/O = C-NH_2_) at a wavelength of 190, 205 or 214 nm [17], [18], [19]. However, the current acceptable measure of protein co-precipitation, during an RNA isolation procedure, is determined by assessing the Abs_260/280_ ratio. The isolated untreated RNA samples fell within the acceptable range of 1.8 to 2.1 (Table 1). Therefore, the isolation procedure employed removed any possible protein contamination (see Data S5).

The interaction of NPs with proteins has recently been reviewed, which explains the effect of NPs on protein stability, solubility etc. [20]. It is tempting to speculate that the shift in the 24 h treated sample was due to the AuNP interacting with residual protein in the RNA sample. However, the Abs_260/280_ ratio clearly disputes the possibility of protein contamination. Other causes of the scattering of UV light, as represented by wavelength shifts at 190 nm must, therefore, be considered. These may include any kind of particulate matter, e.g. non-protein constituents, dust, salts or certain buffer components [21]. Changes to chromophores would also result in wavelength shifts. Due to the helical configuration of nucleotides found in DNA and RNA, the structure causes the chromophores to be closely packed together. Therefore, the chromophores cannot be considered as independent of each other [22]. Any electronic displacement that may occur when one base absorbs quantum energy is actually expressed in the neighboring bases as a modification of the electrical field. Although the total absorption intensity will not necessarily be distributed equally, a shift in the maximum wavelength of absorption may be produced. Intermolecular interactions with the solvents should also be considered. An example includes the hypochromism observed for DNA at the 260 nm band, which is related to conformational changes [23]. The UV bands of the chromophores inherent to nucleic acid structure were indicated in Table 2 showing the wavelength (nm) and the extinction coefficient (*ε*).

It is plausible that the observed wavelength shift in the treated RNA sample could be associated with interactions affecting the amine group or the phosphodiester linkage between the individual nucleotides. AuNPs have a negative surface charge and possess a highly reactive surface, which in turn is able to interact strongly with thiols, disulfides and amines [24], [25]. This indication of structural instability can be interpreted as damage to the RNA molecule. The negatively charged AuNPs used experimentally herein may have interacted with Mg^2+^ metal ions and, thus, created a different kind of structural instability within the RNA molecule. The wavelength shift between the control and the 24 h AuNP treated RNA sample is most likely the result of a combination of all of the above mentioned interactions. The effect of such interactions of NPs on the transcription and/or translation of RNA may, thus, need further investigation. Therefore, studies are currently underway to assess AuNP-induced alterations, if any, related to the amplification of reference genes or other related genes of interest.

DNA has also been assessed in other studies in order to determine the putative effects of NPs with regard to not only the quantity, but also the quality of the nucleic acid. In the first example, it was observed that a dA nucleoside could bind to 13 nm AuNPs via the N(7) nitrogen atom of the imidazole ring [26]. In addition, dC was found to bind the gold surface via a N(3) nitrogen atom of the pyrimidine ring. However, the C = O and C = N bonds in dC can also bind to the gold surface, where the C = O spectral band shifts to a lower frequency due to a decrease in the double bond character, where electron delocalization was induced by coordination of the C = O group on the gold metal surface. The two different nitrogen atoms of the pyrimidine ring and the amino group in dG were also assessed and it was found that the N(1) of the pyrimidine ring had a higher affinity once the hydrogen migrated to the amino group. In contrast, dT could bind to the gold surface via the oxygen of the C(4) = O group of the pyrimidine ring. Further developments have lead to another example where it was found that binding of a functionalized AuNP caused a reversible conformational change in the structure of DNA [27]. Thereafter it was found that a weakly charged, ligand-functionalized AuNP (with a 1.4 nm diameter gold core) could induce plasmid dsDNA to bend and the strands to separate [16]. Another example of the most recent studies showed that a different NP, C_60_, could bind with the minor grooves of dsDNA [28]. This triggered unwinding and disruption of the DNA helix, which could potentially inhibit DNA replication and cause toxic effects at a systemic level. In contrast, the C_60_ NP could bind only to the major grooves of the RNA helix. This transformed the configuration from “stretch” to “curl” and stabilized the RNA structure. Consequently, the examples discussed above emphasize the need for in-depth studies of not only nucleic acid quantity, but also quality before using DNA or RNA as the starting material for genetic studies.

### 3) The fate of AuNP during cell harvesting and RNA isolation

It was expected that a decrease in the UV-Vis absorbance would be observed in RNA isolated from 24 h AuNP-treated BEAS-2B cells (Figure 3C). However, this did not occur, even though the same AuNPs quenched the UV-Vis absorbance in post-isolation spiked samples (Figure 1C). Based on this observation, the fate of the AuNPs was tracked through the eluted flow-through or discarded waste (eluents) from all the wash steps produced (during RNA isolation of BEAS-2B cells treated for 24 h). The AuNPs were found to readily associate with the cell culture media (Figure 7). Therefore, the AuNPs mostly precipitate out in the first eluent containing media only. To a lesser degree, AuNPs were detected in the third step containing cells only, and, the fourth eluent containing media and trypsin. Continuing with the cells, the AuNPs were visibly trapped by the QIAshredder spin column, which is directly after the cell lysis step during the RNA isolation procedure. Thus, during cell harvesting of an adherent cell line, the dark pink/black color observed after treatment with AuNPs actually represented the majority of the AuNPs present in that sample. In addition, the optimized method used to isolate this RNA was also able to prevent AuNP co-precipitation, which was confirmed by CytoViva dark field microscopy (Figure 6).

## Conclusion

The interaction and interference of AuNP with either isolated pure RNA, or, during the isolation procedure was assessed. It was found that the introduction of AuNPs to BEAS-2B cells produced absorbance peak shifts, which indicated changes in the quality of the isolated RNA. Although the RNA isolated from the 24 h AuNP-treated samples was considered to be suitable for RNA-based techniques when using the traditional methods, additional screening identified changes that are associated with structural alterations of functional groups. The observed wavelength shift between the control and the 24 h AuNP-treated RNA (from 190 to 235 nm), could be associated with either the amine, ester or carboxyl compounds based on their typical wavelength absorption. Since protein did not co-precipitate with the RNA during the isolation procedure (evidenced by the Abs_260/280_ ratio), the wavelength shift observed was most probably due to these AuNPs interacting with the amines found in nitrogenous bases of the nucleic acid. However, these AuNPs could have interacted with the phosphodiester linkage between the individual nucleotides, or even the stabilizing Mg^2+^ metal ions within the RNA molecule required for secondary and tertiary structures. It was also found that the artificial introduction of an AuNP (spikes), to a control RNA sample, influenced quantification and purity analyses. It was proposed that the decreased absorbance observed could also occur in other NP-related research, where techniques rely on RNA as the starting material. Caution is, thus, advised when only assessing DNA/RNA quantity, since structurally altered or damaged nucleic acids could be falsely interpreted as simply a low yield and, subsequently, produces false genetic expression data. It was recommended that screening of additional wavelengths other than the traditional 230, 260 and 280 nm would give an indication of RNA quality and purity after treatment with any NP and, subsequently, would identify damage that may have occurred. In addition, it was found that electrophoresis performed on the spiked samples was not sensitive enough to detect changes in the RNA integrity. The fate of the AuNPs was determined by tracking the different eluted flow-through or discarded waste from all the wash steps produced during the RNA isolation procedure. CytoViva dark field microscopy, in conjunction with HSI, was used to confirm that the AuNPs did not co-precipitate in the final product. Despite the lack of co-precipitation of the AuNPs with RNA, structural changes in RNA could still be observed. Future studies will, therefore, aim to assess the effect of AuNP-induced structural changes related to the amplification of reference genes and other related genes of interest. The validation procedure reported herein could be used to develop SOPs and contribute towards developing improved hazard identification of NPs, by acting as a template for assessment of NP interference during the isolation, quantification and integrity analysis of RNA from any human cell line.

## Supporting Information

Data S1 Supporting Figures. Figure S1.1. **The experimental overview.** Figure S1.2. **The RNA isolation procedure indicating where the AuNP spikes were added**
(DOCX)

Data S2 **Supporting Figures and Tables. Figure S2.1.** Absorbance spectrum of the gold nanoparticles. **Figure S2.2.** Characterization of the gold nanoparticles in milli-Q water and in culture medium using Transmission Electron Microscopy. **Table S2.1.** Physicochemical properties of gold nanoparticles in milli-Q water. **Table S2.2.** Physicochemical properties of gold nanoparticles suspended at 1 nM in culture medium.
(DOCX)

Data S3 **Supporting Figures. Figure S3.1.** Fate of AuNP during processing. **Figure S3.2.** Eluent 1. **Figure S3.3.** Eluent 2. **Figure S3.4.** Eluent 4. **Figure S3.5.** Cell pellets and the RNAprotect solution. **Figure S3.6.** Columns used during the RNA isolation, after the cell lysis buffer was added. **Figure S3.7.** Wash solutions used after cell lysis and filtration through the various columns.
(DOCX)

Data S4 **Supporting Tables. Table S4.1.** Purity analysis of untreated control vs. post-isolation AuNP-spiked RNA. **Table S4.2.** Purity analysis of untreated control vs. co-isolation AuNP-spiked RNA.
(DOCX)

Data S5 **Supporting Figures. Figure S5.1.** UV-Vis spectroscopy of a universal RNA standard spiked with AuNP. **Figure S5.2.** UV-Vis spectroscopy of a universal RNA standard spiked with AuNP. (A) First technical repeat. (B) Second technical repeat.
(DOCX)
