# Supplementary material for: Gold Nanoparticle Interference Study during the Isolation, Quantification, Purity and Integrity Analysis of RNA
Source: PLoS One. 2014 Dec 3;9(12):e114123. doi: 10.1371/journal.pone.0114123 (PMC4254911; doi:10.1371/journal.pone.0114123)
Supplement: Data S1 — Supporting Figures. Figure S1.1. The experimental overview. Figure S1.2. The RNA isolation procedure indicating where the AuNP spikes were added (DOCX) [file pone.0114123.s001.docx]

**Title:** Gold nanoparticle interference study during the isolation, quantification, purity and integrity analysis of RNA.

**Authors:** NM Sanabria, M Vetten, C Andraos, K Boodhia, M Gulumian

***Supplementary Data 1:* Experiment Overview**


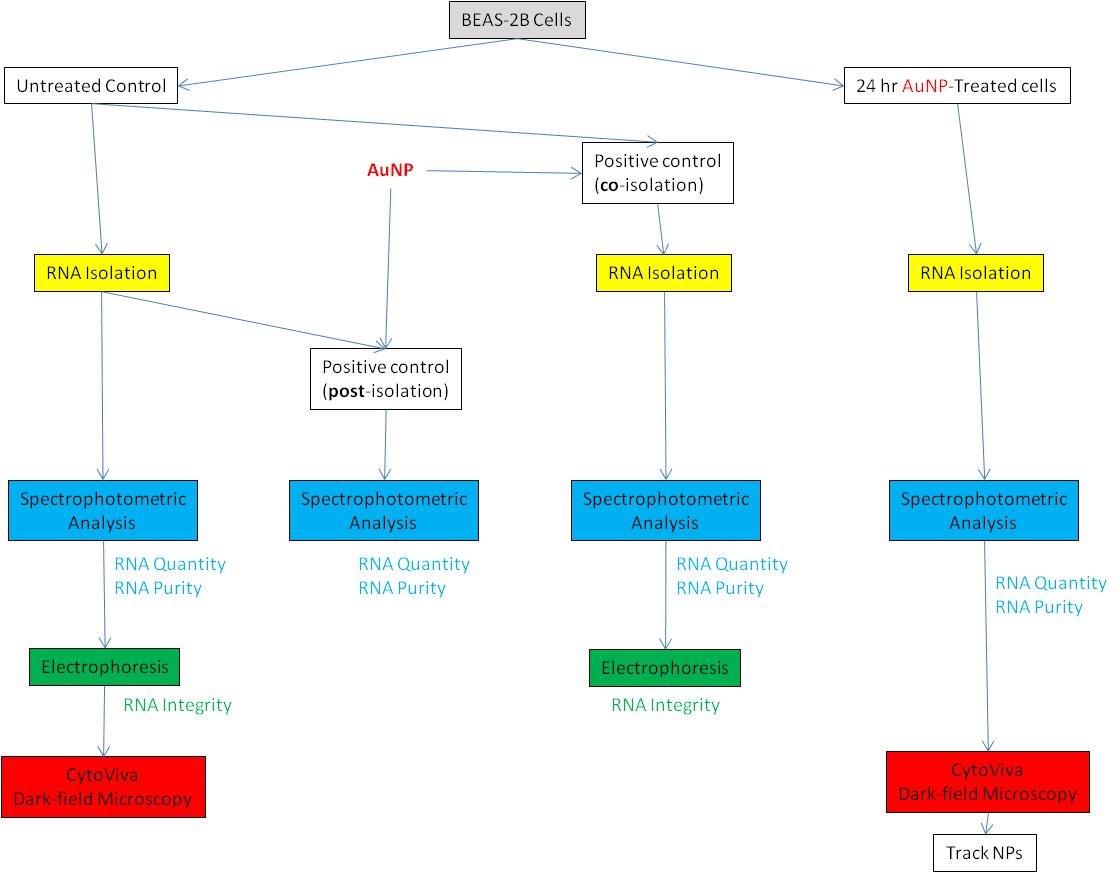


**Figure S1.1:** **The experimental overview.**


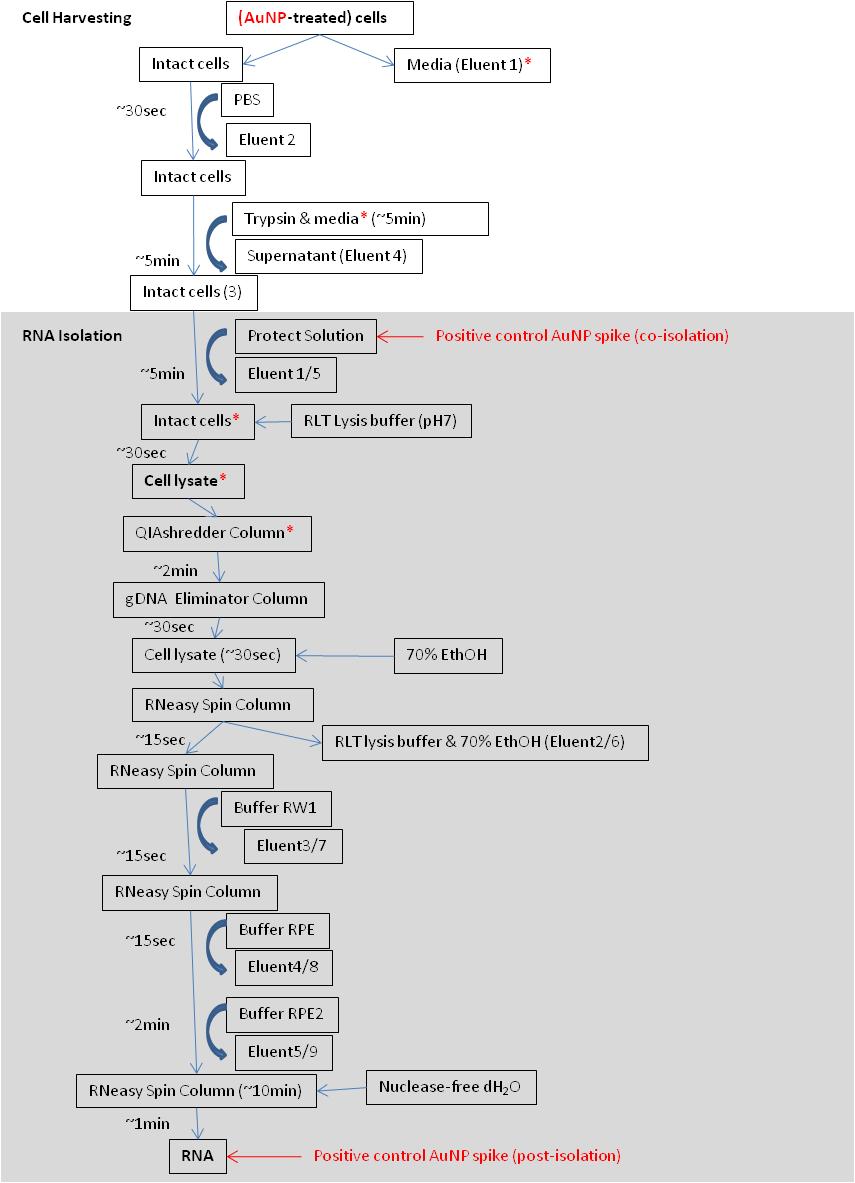


**Figure S1.2:** **The RNA isolation procedure indicating where the AuNP spikes were added.**
