# Supplementary material for: Gold Nanoparticle Interference Study during the Isolation, Quantification, Purity and Integrity Analysis of RNA
Source: PLoS One. 2014 Dec 3;9(12):e114123. doi: 10.1371/journal.pone.0114123 (PMC4254911; doi:10.1371/journal.pone.0114123)
Supplement: Data S2 — Supporting Figures and Tables. Figure S2.1. Absorbance spectrum of the gold nanoparticles. Figure S2.2. Characterization of the gold nanoparticles in milli-Q water and in culture medium using Transmission Electron Microscopy. Table S2.1. Physicochemical properties of gold nanoparticles in milli-Q water. Table S2.2. Physicochemical properties of gold nanoparticles suspended at 1 nM in culture medium. (DOCX) [file pone.0114123.s002.docx]

**Title:** Gold nanoparticle interference study during the isolation, quantification, purity and integrity analysis of RNA.

**Authors:** NM Sanabria, M Vetten, C Andraos, K Boodhia, M Gulumian

***Supplementary Data 2:* AuNP Characterisation**

This AuNP was fully characterised and the results published (Vetten *et al.,* 2013). Table S2.1 shows the physicochemical properties of the AuNPs in milli-Q H2O. The surface charge of the particles, as described by their zeta (ζ) potential, are all negatively charged and of similar value. Table S2.2 shows the physicochemical properties of the AuNPs after centrifugation and re-suspension in both RPMI and Ham’s-F12 culture medium. The pH of the RPMI and Ham’s-F12 culture medium at 37°C are pH 7.66 and 7.96 respectively. In all instances, the pH of the culture medium decreased slightly when the particles were re-suspended therein; however the observed decreases were not biologically significant. The particles dispersed in Ham’s F12 culture medium showed a broader peak as compared to those in RPMI culture medium and water (Table S2.2 and Figure S2.1), owing to the difference in the refractive index of the medium and the final composition. AuNPs were further characterised with TEM and the representative images are shown in Figure S2.2. The TEM data demonstrates that the integrity of the AuNPs suspended in cell culture medium was maintained as compared to the AuNPs in water, with minimal signs of particle aggregation present. The AuNPs were primarily spherical in shape, with some ellipsoid nanoparticles present, probably due to synthetic conditions employed as factors such as salt concentration, pH, and temperature during synthesis have been shown to affect the polydispersity of AuNPs (Li *et al.,* 2011; Zabetakis *et al.,* 2012).

**Table S2.1**: **Physicochemical properties of gold nanoparticles in milli-Q water** (Vetten *et al.,* 2013).

| **Nanoparticle** | **Particle size determined by TEM** | **ζ-potential** | **Citrate concentration** | **UV–vis** | **Particles/ml (at 1 nM)** |
| --- | --- | --- | --- | --- | --- |
| 14 nm AuNP | 14 ± 2 nm | −33.5 mV | 0.025% | 520 nm | 2.25 x 10^12^ nps/ml |
| 20 nm AuNP | 20 ± 2 nm | −37.9 mV | 0.023% | 524 nm | 7.76 x 10^11^ nps/ml |

**Table S2.2**: **Physicochemical properties of gold nanoparticles suspended at 1 nM in culture medium** (Vetten *et al.,* 2013).

| Nanoparticle | ζ- Potential (mV) | | pH at 37°C | | Absorbance peak | | Average particle size in nm  determined by TEM (Std Dev) | |
| --- | --- | --- | --- | --- | --- | --- | --- | --- |
|  | RPMI | Ham’s-F12 | RPMI (pH 7.66) | Ham’s-F12 (pH 7.96) | RPMI | Ham’s-F12 | RPMI | Ham’s-F12 |
| 14 nm AuNP | −10.24 | −11.40 | 7.61 | 7.84 | 557 nm | 535 nm | 17 nm (± 3 nm) | 16 nm (± 3 nm) |
| 20 nm AuNP | −12.06 | −11.30 | 7.61 | 7.87 | 557 nm | 532 nm | 22 nm (± 4 nm) | 24 nm (± 3 nm) |

**Figure S2.1**: **Absorbance spectrum of the gold nanoparticles**. The UV–vis spectrum of 14 nm and 20 nm AuNPs at 1 nM in, A) milli-Q water, B) in RPMI culture medium, and C) in Ham’s F12 culture medium (Vetten *et al*., 2013).

**Figure S2.2:** **Characterisation of the gold nanoparticles in milli-Q water and in culture medium using Transmission Electron Microscopy**. TEM images of 14 nm AuNPs in (A) water, (B) RPMI culture medium, and (C) Ham’s F12 culture medium; and 20 nm AuNPs in (D) water, (E) RPMI culture medium, and (F) Ham’s F-12 culture medium (Vetten *et al*., 2013).

**References:**

Vetten MA, Tlotleng N, Rascher DT, Skepu A, Keter FK, Boodhia K, Koekemoer L-A, Andraos C, Tshikhudo R, Gulumian M (2013) Label-free in vitro toxicity and uptake assessment of citrate stabilised gold nanoparticles in three cell lines. *Particle and Fibre Toxicology* **10**:50.

Li C, Li D, Wan G, Xu J, Hou W (2011) Facile synthesis of concentrated gold nanoparticles with low size-distribution in water: temperature and pH controls. *Nanoscale Res Lett*. **6**:440.

Zabetakis K, Ghann WE, Kumar S, Daniel M-C (2012) Effect of high gold salt concentrations on the size and polydispersity of gold nanoparticles prepared by an extended Turkevich-Frens method. *Gold Bull*. **45**:203–211.

______________________________________________________________
