# Supplementary material for: Gold Nanoparticle Interference Study during the Isolation, Quantification, Purity and Integrity Analysis of RNA
Source: PLoS One. 2014 Dec 3;9(12):e114123. doi: 10.1371/journal.pone.0114123 (PMC4254911; doi:10.1371/journal.pone.0114123)
Supplement: Data S3 — Supporting Figures. Figure S3.1. Fate of AuNP during processing. Figure S3.2. Eluent 1. Figure S3.3. Eluent 2. Figure S3.4. Eluent 4. Figure S3.5. Cell pellets and the RNAprotect solution. Figure S3.6. Columns used during the RNA isolation, after the cell lysis buffer was added. Figure S3.7. Wash solutions used after cell lysis and filtration through the various columns. (DOCX) [file pone.0114123.s003.docx]

**Title:** Gold nanoparticle interference study during the isolation, quantification, purity and integrity analysis of RNA.

**Authors:** NM Sanabria, M Vetten, C Andraos, K Boodhia, M Gulumian

***Supplementary Data 3:* The fate of the AuNPs**

The expected decrease of the traditional UV-Vis absorbance (220-340 nm) was not observed in RNA isolated from 24 h AuNP-treated BEAS-2B cells. However, a spiked sample (positive control) did prove that a decrease of the UV-Vis absorbance was caused by AuNPs. Therefore, the fate of the AuNPs was tracked during cell harvesting of this adherent cell line. Firstly, the cells were separated from the growth media and then washed with PBS, before being trypsinized. Thereafter, the cells were precipitated by centrifugation for harvesting. The RNA isolation procedure that followed also included wash and centrifugation steps. Since the citrate capped AuNP has a UV absorbance at 519 nm, this was the initial wavelength chosen for analyses. The NPs were tracked through the eluted flow-through or discarded waste (eluents) from all the wash steps produced during the RNA isolation procedure. The AuNPs were found to readily associate with the cell culture media. Therefore, the AuNPs precipitated out in the first “media-only” eluent, the third sample of “cells only” and the fourth eluent of “media and trypsin”. Continuing with the cells (sample 3), the AuNPs were visually observed to be trapped by the QIAshredder spin column.

**
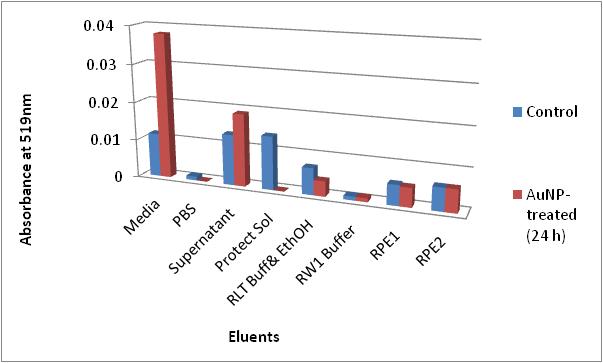
**

**Figure S3.1**: **Fate of AuNP during processing.** All samples were measured at 519 nm, against a blank of nuclease-free water.

1. ***Cell harvesting:***

RNA was isolated from 24 h AuNP-treated BEAS-2B cells. During cell harvesting of an adherent cell line (BEAS-2B), the cells are first separated from the growth media, which was dark pink/black in colour after treatment with NPs. The cells were then washed with PBS, before being trypsinized. The cells were finally collected by centrifugation. The eluents obtained from harvesting cells from adherent cell line BEAS-2B were as follows; (i) The cell culture growth media (eluent 1) that is removed from the culture flasks, before cell harvesting, (ii) PBS (eluent 2) used to wash the adherent cells in the culture flasks, before trypsin digest, (iii) The actual cells / pellet (sample 3) used for RNA isolation, which was separated from the supernatant, (iv) The supernatant obtained (eluent 4), after cells (sample 3) were precipitated via centrifugation. It is important to note that the media (eluent 1) was removed during cell harvesting, before the cell lysis step that is required in the RNA isolation procedure, i.e. most of the AuNP was not present later on when cell lysis occurred. Therefore, very little AuNP was in contact with the exposed RNA. The PBS wash step was also performed during the cell collection, i.e. before the cell lysis step. In addition, the supernatant was separated from the cells before the lysis step.

**(A) (B)**


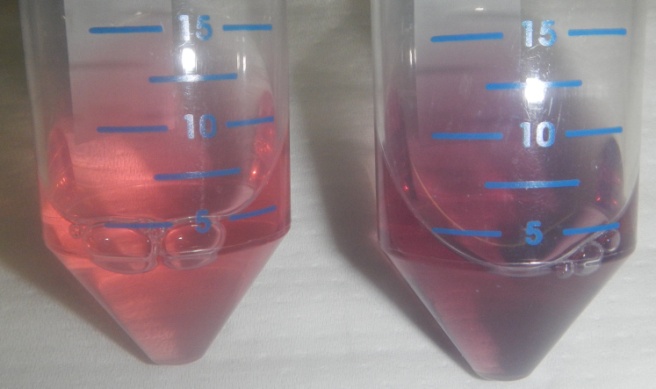

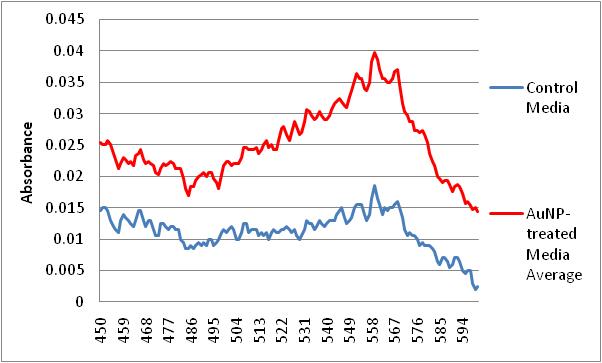


**Figure S3.2: Eluent 1. (A)** Media collected from untreated control (LHS) and AuNP-treated (RHS). **(B)**The average absorbance reading obtained for 3 replicates, for 1 µl of media from the cell culture flasks. All samples were measured against a blank of nuclease-free water.

**(A) (B)**


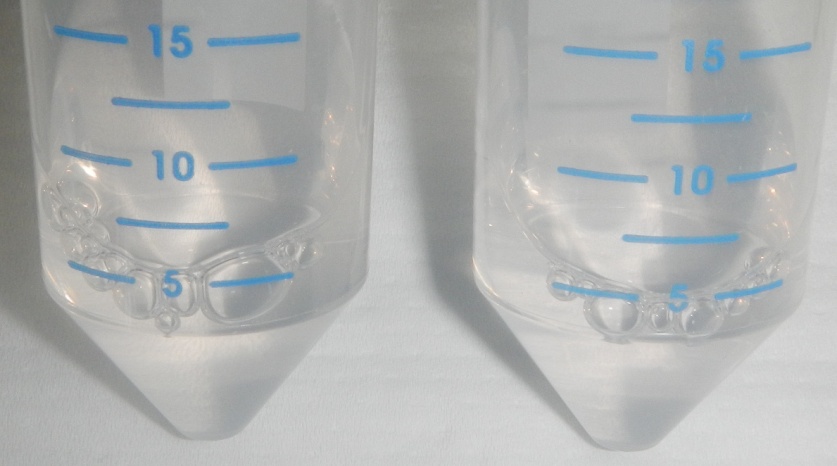

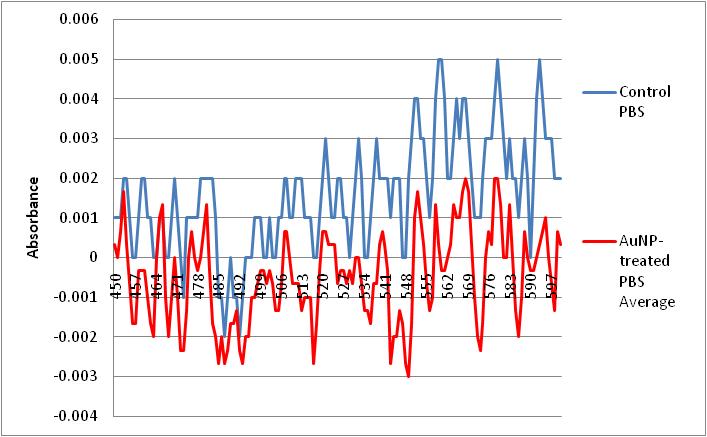


**Figure S3.3: Eluent 2. (A)** PBS collected from untreated control (LHS) and AuNP-treated (RHS).

**(B)** The average absorbance reading obtained for 3 replicates, for 1 µl of PBS obtained from the first wash of the cell cultures. All samples were measured against a blank of nuclease-free water.

**(A) (B)**


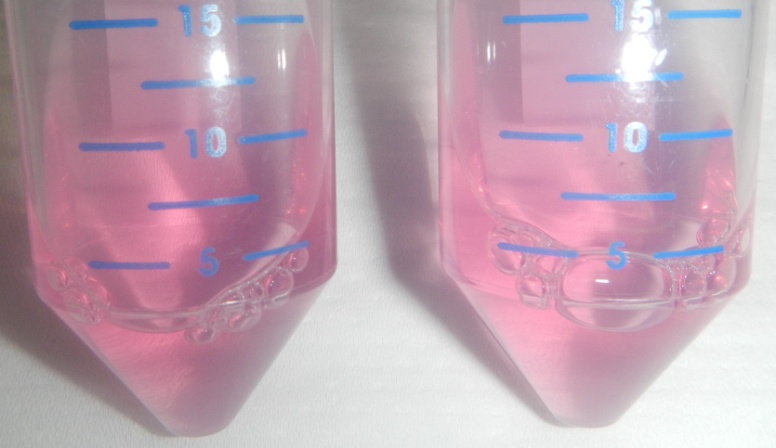

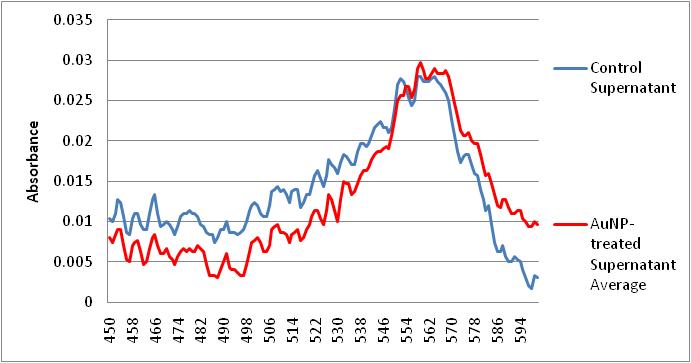


**Figure S3.4: Eluent 4. (A)** Supernatant consisting of media and trypsin, which was collected from untreated control (LHS) and AuNP-treated (RHS). **(B)** The average absorbance reading obtained for 3 replicates, for 1 µl of supernatant after the cells were centrifuged and removed. All samples were measured against a blank of nuclease-free water.

1. ***RNA isolation using RNAprotect®***

The cells were collected by centrifugation during the harvesting. The RNA isolation procedure that followed was initiated by re-suspending this pellet in an RNAprotect® solution, in order to protect the RNA during extended handling time frames or storage. Therefore, the RNAprotect® solution must be removed again immediately before processing the sample any further. Once the solution had been removed, the cells were treated with an RLT Lysis buffer. This buffer contained guanidine thiocyanate and β-mercaptoetghanol, at pH 7. The sample was then processed through the QIAshredder spin column. It was visibly observed that the AuNPs, from the treated sample, were trapped by the QIAshredder spin column. The total number of eluents obtained from the RNA isolation procedure whilst using RNAprotect®, QIAshredder columns and the RNeasy Plus kit were as follows; (i) The cell pellet (sample 3) as suspended in an RNAprotect® solution, (ii) After the cells were removed, the remaining RNAprotect® supernatant represented eluent 1 of the RNA isolation, but eluent 5 of the entire procedure, (iii) RLT lysis buffer combined with 70% EthOH represented eluent 2 of the isolation, but eluent 6 of the entire procedure, (iv) Buffer RW1 represented eluent 3 of the isolation, but eluent 7 of the entire procedure, (v) Buffer RPE represented eluent 4 of the isolation, but eluent 8 of the entire procedure, (vi) Buffer RPE2 represented eluent 5 of the isolation, but eluent 9 of the entire procedure.

**(A) (B)**


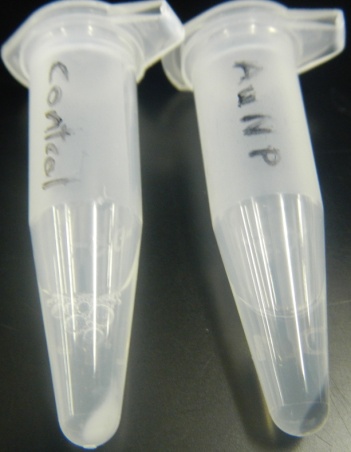

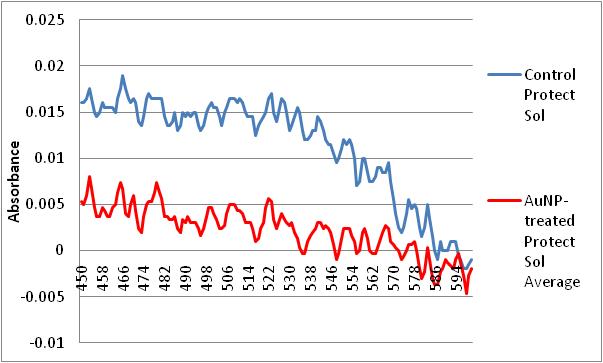


**Figure S3.5:** **Cell pellets and the RNAprotect® solution. (A)** Cells were collected from untreated control (LHS) and AuNP-treated (RHS) samples by centrifugation. **(B)** The average absorbance reading obtained for 3 replicates, for 1 µl of Protect solution after the cells had been removed for further processing. All samples were measured against a blank of nuclease-free water.

**(A) (B)**


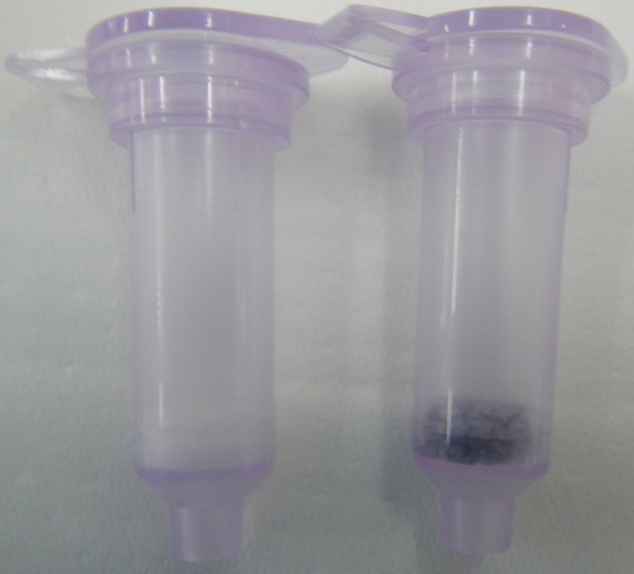

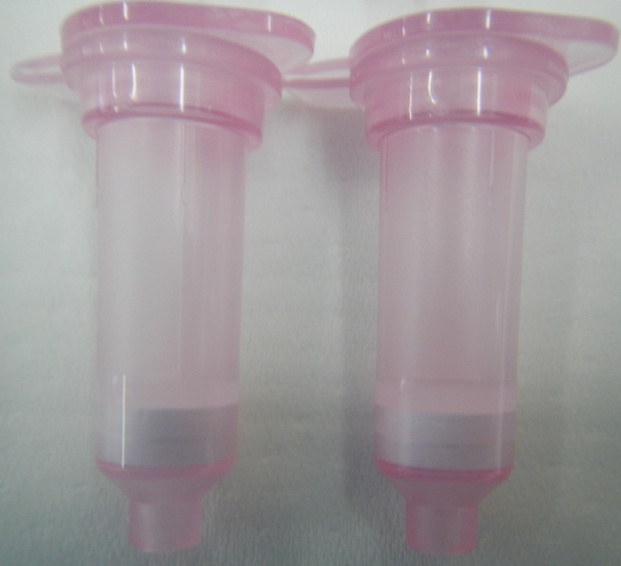


**Figure S3.6: Columns used during the RNA isolation, after the cell lysis buffer was added. (A)** Cell debris and AuNPs trapped on QIAshredder column, from untreated control (LHS) and AuNP-treated (RHS) samples. **(B)** Cell lysates are passed through the RNeasy spin columns, from untreated control (LHS) and AuNP-treated (RHS) samples.

**(A) (B)**


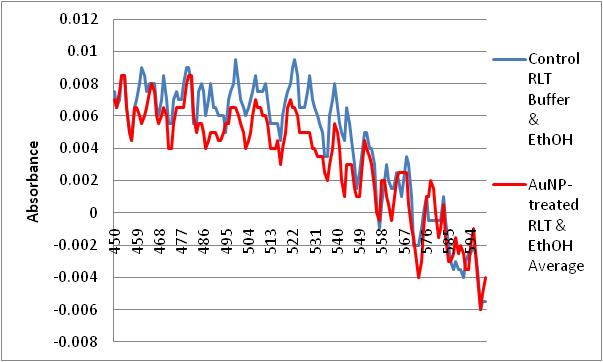

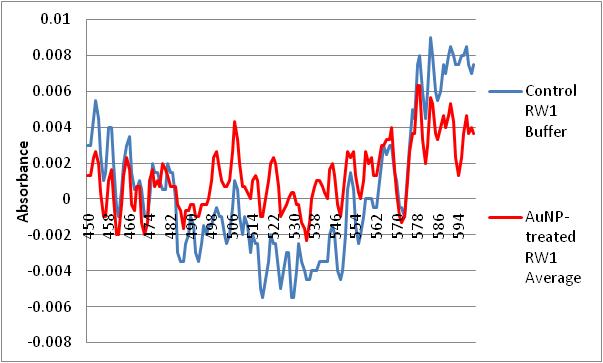


**(C) (D)**


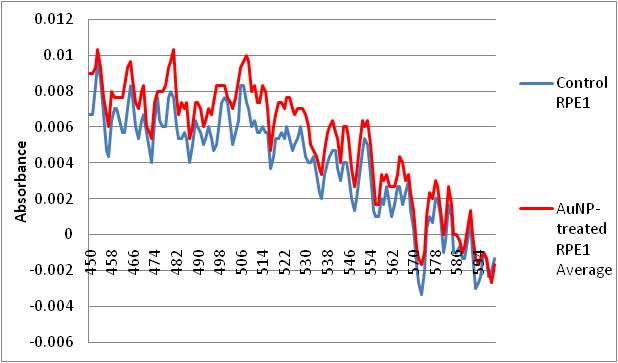

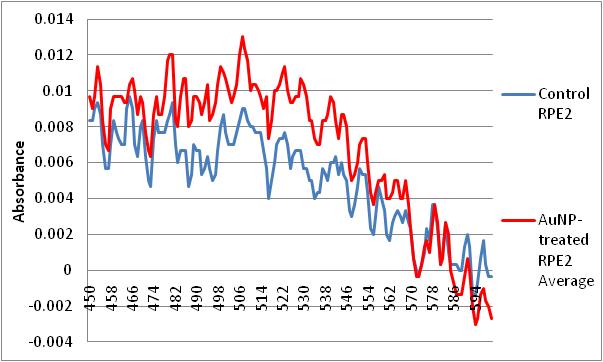


**Figure S3.7: Wash solutions used after cell lysis and filtration through the various columns. (A)** The average absorbance reading obtained for 3 replicates, for 1 µl of the RLT buffer mixed with 70% ethanol, which was the discarded flow-through from the QIA-shredder, gDNA-Eliminator and RNeasy spin columns. **(B)** The average absorbance reading obtained for 3 replicates, for 1 µl of the RW1 Buffer, which was the discarded flow-through from the first wash of the RNeasy spin column. **(C)** The average absorbance reading obtained for 3 replicates, for 1 µl of the RPE Buffer, which was the discarded flow-through. **(D)** The average absorbance reading obtained for 3 replicates, for 1 µl of the RPE2 Buffer, which was the discarded flow-through.
