# Supplementary material for: Gold Nanoparticle Interference Study during the Isolation, Quantification, Purity and Integrity Analysis of RNA
Source: PLoS One. 2014 Dec 3;9(12):e114123. doi: 10.1371/journal.pone.0114123 (PMC4254911; doi:10.1371/journal.pone.0114123)
Supplement: Data S4 — Supporting Tables. Table S4.1. Purity analysis of untreated control vs. post-isolation AuNP-spiked RNA. Table S4.2. Purity analysis of untreated control vs. co-isolation AuNP-spiked RNA. (DOCX) [file pone.0114123.s004.docx]

**Title:** Gold nanoparticle interference study during the isolation, quantification, purity and integrity analysis of RNA.

**Authors:** NM Sanabria, M Vetten, C Andraos, K Boodhia, M Gulumian

***Supplementary Data 4:* Purity analysis of spiked samples**

**Table S4.1: Purity analysis of untreated control vs. post-isolation AuNP-spiked RNA**

| **Sample ID** | **A_260_** | **A_280_** | **A_260_/A_280_** | **A_260_/A_230_** | **Purity Analysis** | **True/False Quantification** |
| --- | --- | --- | --- | --- | --- | --- |
| Control A | 6.471 | 3.123 | 2.07 | 1.70 | Good | True |
| Control A+**25%**NP | 5.044 | 2.456 | 2.05 | 1.44 | Reduced yield, no protein contamination, but contaminants are present | False |
| Control A +**50%**NP | 3.540 | 1.746 | 2.03 | 1.29 | Reduced yield, no protein contamination, but contaminants are present | False |
| Control A+**75%**NP | 2.996 | 1.494 | 2.00 | 1.54 | Reduced yield, no protein contamination, no contaminates | False |
| Control B | 23.116 | 11.106 | 2.08 | 1.47 | Good | True |
| Control B +**25%**NP | 18.571 | 8.864 | 2.10 | 1.43 | Reduced yield, no protein contamination, but contaminants are present | False |
| Control B +**50%**NP | 13.120 | 6.207 | 2.11 | 1.40 | Reduced yield, no protein contamination, but contaminants are present | False |
| Control B l+**75%**NP | 8.082 | 3.927 | 2.06 | 1.40 | Reduced yield, no protein contamination, but contaminants are present | False |
| Control C | 3.309 | 1.646 | 2.01 | 1.28 | Relatively good, where no protein contamination occurred, but contaminants are present | True |
| Control C +**25%**NP | 3.032 | 1.511 | 2.01 | 1.23 | Reduced yield, no protein contamination, but contaminants are present | False |
| Control C +**50%**NP | 2.033 | 1.038 | 1.96 | 1.31 | Reduced yield, no protein contamination, but contaminants are present | False |
| Control C +**75%**NP | 1.849 | 0.945 | 1.96 | 1.13 | Reduced yield, no protein contamination, but contaminants are present | False |
| Control A & ***Protect*** sol | 4.757 | 2.333 | 2.04 | 1.98 | Good | True |
| Control A & ***Protect*** sol +**25%**NP | 3.351 | 1.675 | 2.00 | 1.56 | Reduced yield, with no protein contamination and no contaminates | Improvement, but still false |
| Control A & ***Protect*** sol +**50%**NP | 2.643 | 1.319 | 2.00 | 1.95 | Reduced yield, with no protein contamination and no contaminates | Improvement, but still false |
| Control A & ***Protect*** sol +**75%**NP | 1.725 | 0.886 | 1.95 | 1.62 | Reduced yield, with no protein contamination and no contaminates | Improvement, but still false |
| Control B & ***Protect*** sol | 18.622 | 8.952 | 2.08 | 1.88 | Good | True |
| Control B & ***Protect*** sol +**25%**NP | 15.523 | 7.420 | 2.09 | 1.92 | Reduced yield, with no protein contamination and no contaminates | Improvement, but still false |
| Control B & ***Protect*** sol +**50%**NP | 11.884 | 5.704 | 2.08 | 1.91 | Reduced yield, with no protein contamination and no contaminates | Improvement, but still false |
| Control B & ***Protect*** sol +**75%**NP | 6.993 | 3.424 | 2.04 | 1.82 | Reduced yield, with no protein contamination and no contaminates | Improvement, but still false |
| Control C & ***Protect*** sol | 13.253 | 6.648 | 1.99 | 1.89 | Good | True |
| Control C & ***Protect*** sol +**25%**NP | 8.926 | 4.338 | 2.06 | 1.75 | Reduced yield, with no protein contamination and no contaminates | Improvement, but still false |
| Control C & ***Protect*** sol +**50%**NP | 7.607 | 3.669 | 2.07 | 1.75 | Reduced yield, with no protein contamination and no contaminates | Improvement, but still false |
| Control C & ***Protect*** sol +**75%**NP | 4.895 | 2.370 | 2.07 | 1.85 | Reduced yield, with no protein contamination and no contaminates | Improvement, but still false |

**Table S4.2: Purity analysis of untreated control vs. co-isolation AuNP-spiked RNA**

| **Sample ID** | **A_260_** | **A_280_** | **A_260_/A_280_** | **A_260_/A_230_** | **Purity Analysis** | **True/False Quantification** |
| --- | --- | --- | --- | --- | --- | --- |
| Control A | 2.599 | 1.284 | 2.02 | 0.82 | Good, but with isolation contaminates. | True |
| Control A+**25 µl** NP | 13.016 | 6.299 | 2.07 | 2.15 | Increased yield, with no protein contamination and no isolation contaminates | False |
| Control A +**50µl** NP | 12.703 | 6.301 | 2.02 | 1.87 | Increased yield, with no protein contamination and no isolation contaminates | False |
| Control A+**100µl** NP | 9.295 | 4.483 | 2.07 | 2.25 | Increased yield, with no protein contamination and no isolation contaminates | False |
| Control B | 2.775 | 1.363 | 2.04 | 0.81 | Good, but with isolation contaminates. | True |
| Control B+**25µl** NP | 13.199 | 6.425 | 2.05 | 2.13 | Increased yield, with no protein contamination and no isolation contaminates | False |
| Control B +**50µl** NP | 12.421 | 6.035 | 2.06 | 1.87 | Increased yield, with no protein contamination and no isolation contaminates | False |
| Control B+**100µl** NP | 9.450 | 4.578 | 2.06 | 2.16 | Increased yield, with no protein contamination and no contaminates | False |
| Control C | 23.99 | 11.42 | 2.10 | 1.48 | Good | True |
| Control C+**25 µl** NP | 33.084 | 15.819 | 2.09 | 1.87 | Increased yield, with no protein contamination and no isolation contaminates | False |
| Control C +**50 µl** NP | 19.294 | 9.232 | 2.09 | 1.67 | Reduced yield, with no protein contamination and no isolation contaminates | False |
| Control C+**100 µl** NP | 20.571 | 9.868 | 2.08 | 1.93 | Reduced yield, with no protein contamination and no isolation contaminates | False |
| Control A | 13.76 | 6.529 | 2.11 | 1.58 | Good | True |
| Control A & ***Protect*** +**25 µl** NP | 12.404 | 6.064 | 2.05 | 1.96 | Reduced yield, with no protein contamination and no isolation contaminates | Improvement, but still false |
| Control A & ***Protect*** +**50 µl**NP | 10.326 | 5.011 | 2.06 | 2.04 | Reduced yield, with no protein contamination and no isolation contaminates | Improvement, but still false |
| Control A & ***Protect*** +**100 µl** NP | 13.514 | 6.584 | 2.05 | 1.47 | Similar yield, with no protein contamination and no isolation contaminates | Improvement |
| Control B & ***Protect*** | 13.96 | 6.577 | 2.12 | 1.65 | Good | True |
| Control B & ***Protect*** +**25 µl** NP | 12.58 | 6.090 | 2.07 | 1.88 | Reduced yield, with no protein contamination and no isolation contaminates | Improvement, but still false |
| Control B & ***Protect*** +**50 µl**NP | 10.416 | 5.064 | 2.06 | 2.03 | Reduced yield, with no protein contamination and no isolation contaminates | Improvement, but still false |
| Control B & ***Protect*** +**100 µl** NP | 12.779 | 6.209 | 2.06 | 1.52 | Reduced yield, with no protein contamination and no isolation contaminates | Improvement, but still false |
| Control C & ***Protect*** | 18.82 | 9.019 | 2.09 | 1.96 | Good | True |
| Control C & ***Protect*** +**25 µl** NP | 15.30 | 7.364 | 2.08 | 1.05 | Reduced yield, with no protein contamination, but with isolation contaminates | Improvement, but still false |
| Control C & ***Protect*** +**50 µl** NP | 17.043 | 7.970 | 2.14 | 2.01 | Reduced yield, possible protein, but no isolation contaminates | Improvement, but still false |
| Control C & ***Protect*** +**100 µl** NP | 16.975 | 7.994 | 2.12 | 1.74 | Reduced yield, possible protein, but no isolation contaminates | Improvement, but still false |
