# Supplementary material for: Gold Nanoparticle Interference Study during the Isolation, Quantification, Purity and Integrity Analysis of RNA
Source: PLoS One. 2014 Dec 3;9(12):e114123. doi: 10.1371/journal.pone.0114123 (PMC4254911; doi:10.1371/journal.pone.0114123)
Supplement: Data S5 — Supporting Figures. Figure S5.1. UV-Vis spectroscopy of a universal RNA standard spiked with AuNP. Figure S5.2. UV-Vis spectroscopy of a universal RNA standard spiked with AuNP. (A) First technical repeat. (B) Second technical repeat. (DOCX) [file pone.0114123.s005.docx]

**Title:** Gold nanoparticle interference study during the isolation, quantification, purity and integrity analysis of RNA.

**Authors:** NM Sanabria, M Vetten, C Andraos, K Boodhia, M Gulumian

***Supplementary Data 5:* Protein contamination investigated for causing wavelength shifts**

The current acceptable measure of protein co-precipitation, during an RNA isolation procedure, is determined by assessing the Abs_260/280_ ratio. Experimentally it was found that the isolated untreated RNA samples fell within the acceptable range of 1.8 to 2.1 (refer to Table 1 in the main text). Therefore, the isolation procedure employed removed any possible protein contamination.

In order to validate the lack of protein contamination in wavelength shifted samples, an additional experiment was conducted, which included the use a bought universal RNA standard (Agilent, USA). The universal RNA standard, usually intended for qPCR use, consisted of total RNA from 10 different human cell lines. This standard had been tested using electrophoresis on 1xMOPS agarose gel, as well as the Agilent 2100 Bioanalyzer and the RNA 6000 Nano LabChip kit.

The universal RNA standard was also spiked with AuNPs (as described in the main text for the samples with a constant amount of RNA) and tested. The results are shown below, where a slight shift in wavelength was still observed between 195 and 200 nm, e.g. for the 50% and 75% AuNP spiked samples. Therefore, protein contamination obtained from the isolation procedure described in the manuscript would not account for the wavelength shifts observed in the bought RNA standard that had already been isolated and purified.


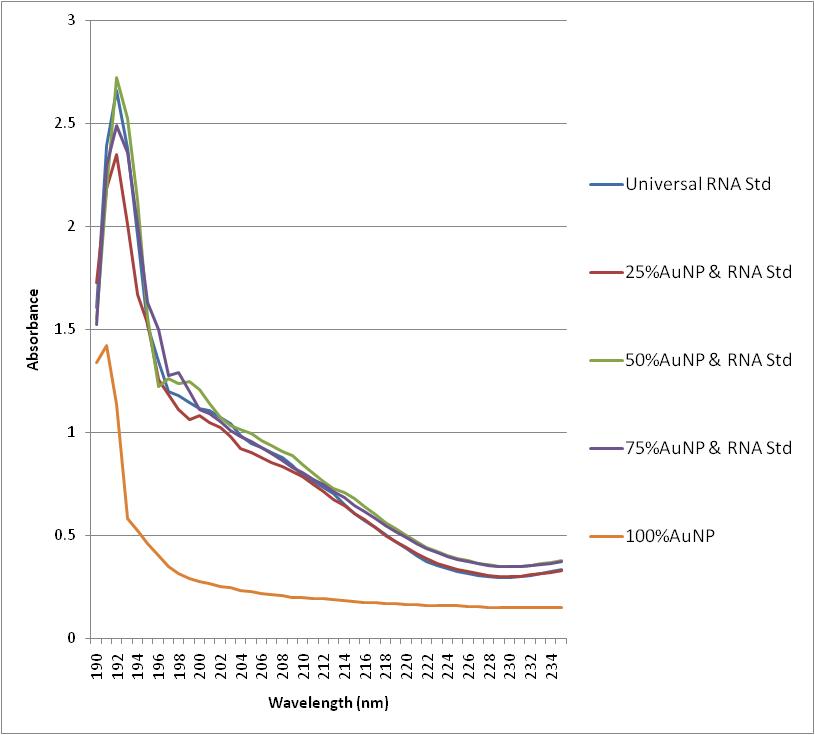


**Figure S5.1**: **UV-Vis spectroscopy of the universal RNA standard spiked with AuNP.**

**A) B)**

**
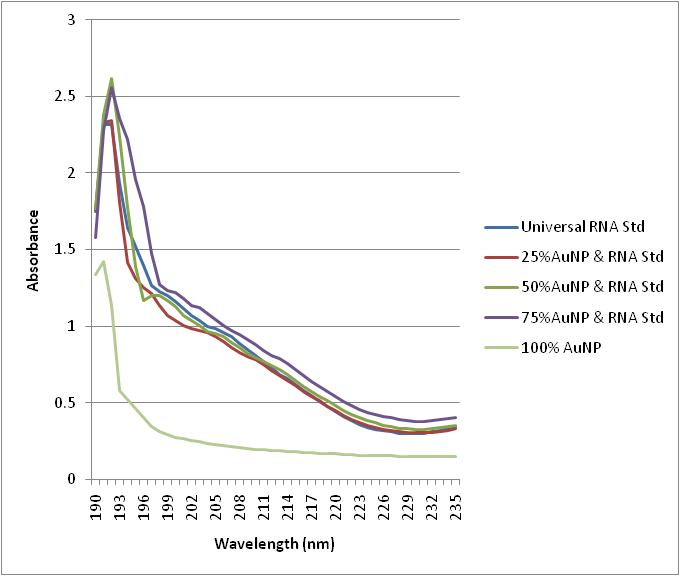

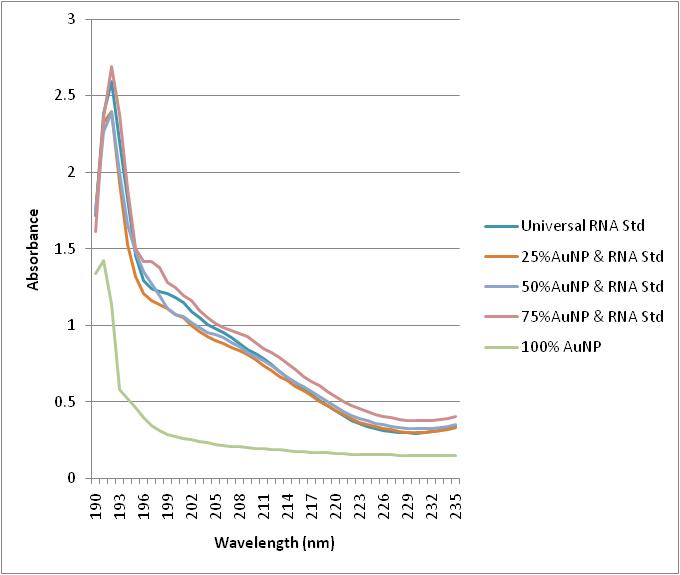
**

**Figure S5.2**: **UV-Vis spectroscopy of the universal RNA standard spiked with AuNP. (A) First technical repeat. (B) Second technical repeat.**
